# Self-DNA mediates tobacco-induced lung inflammation via macrophage-intrinsic cGAS/STING/TBK1 signaling

**DOI:** 10.1186/s12964-026-03124-3

**Published:** 2026-08-10

**Authors:** Hongmei Tang, Linlin Guo, Zhibin Wang, Kaijin Wang, Ziling Zeng, Xiaoyun Wang, Yuejiao Li, Xiefang Yuan, Guofeng Xu, Jiayao Duan, Hang Hu, Yun Zhang, Ning Ma, Xing Wang

**Affiliations:** 1 Inflammation & Allergic Diseases Research Unit, The Affiliated Hospital of Southwest Medical University, Luzhou, 646000 China; 2 Department of Microbiology and Immunology, Indiana University School of Medicine, Indianapolis, 46202 USA; 3 Department of Respiratory and Critical Care Medicine, Bishan Hospital of Chongqing, Bishan Hospital of Chongqing Medical University, Chongqing, 402760 China; 4 Department of Respiratory and Critical Care Medicine, The Affiliated Hospital of Southwest Medical University, Luzhou, 646000 China

**Keywords:** cGAS, STING, Cigarette smoke, mtROS, mtDNA, TBK1, Macrophage, Inflammation

## Abstract

Cigarette smoke is a primary risk factor for chronic obstructive pulmonary disease (COPD). While self-DNA sensors coordinated with the immune system are known to contribute to pollutant-induced responses, the exact mechanisms by which dsDNA drives COPD pathogenesis remain unclear. Here, we demonstrated that dsDNA levels are elevated and cGAS/STING/TBK1 signaling is activated in patients with COPD. In-depth investigations using mice models of COPD, including macrophage-specific *Lyz2*^*cre+*^*Cgas*^*fl/fl*^^,^* Sting-deficient*, and *Lyz2*^*cre+*^*Tbk1*^*fl/fl*^ mice, indicated that cigarette smoke extract (CSE) induces mitochondrial reactive oxygen species (mtROS), leading to the overproduction and release of dsDNA and mitochondrial DNA (mtDNA). Furthermore, we found that activation of the downstream IRF3/NF-κB pathway via cGAS/STING/TBK1 signaling promotes the expression of CD80, CD86, and iNOS, thereby driving M1 macrophage polarization. This pathway also mediates the overexpression of type I interferons and related pro-inflammatory cytokines under CSE exposure. Interestingly, released dsDNA further exacerbates these CSE-induced effects by promoting a feedback loop of mtROS overproduction and additional mtDNA release. Notably, blocking cGAS/STING/TBK1 signaling, inhibiting dsDNA release or decreasing mtROS production significantly attenuates M1 pacrophage polarization. Taken together, our findings reveal that the cGAS/STING/TBK1 pathway acts as a critical mediator of dsDNA/mtDNA-driven lung inflammation by regulating M1 macrophage polarization in response to cigarette smoke. Targeting cGAS/STING/TBK1 signaling axis or its associated mtROS/mtDNA networks might offer novel therapeutic strategies against inflammatory lung diseases caused by environmental hazards.

**Supplementary Information:**

The online version contains supplementary material available at https://doi.org/10.1186/s12964-026-03124-3.

## Introduction

Environmental pollution severely impacts human health. Harmful airborne particles, such as cigarette smoke, are recognized globally as major contributors to cardiovascular disease, various cancers, and chronic lung inflammatory diseases, including chronic obstructive pulmonary disease (COPD) [1–5]. COPD is characterized by airway obstruction associated with tissue remodeling and cellular inflammation [2, 6]. Consequently, modulating inflammation and detecting relevant biomarkers are critical for early diagnosis and the development of effective therapeutic strategies.

Mitochondria serve as central hubs for the generation of ATP and reactive oxygen species [7], while concurrently regulating the development of inflammation [8–10]. Consequently, macrophages with impaired mitochondrial function can drive inflammatory pathogenesis [11]. Emerging evidence heavily links self-DNA signaling to lung inflammation [12]. While various dsDNA sensors, including cGAS, STING, absent in melanoma 2 (AIM2), interferon gamma-inducible 16 (IFI16), Toll-like receptor 9 (TLR9) and DDX41, have well-characterized roles in immune response [13], cGAS stands out as a critical sensor. It detects not only exogenous cytosolic DNA but also endogenous DNA (mtDNA) released into the cytosol [14, 15].

Recent studies indicate that dsDNA release plays a pivotal role in the pathogenesis of lung diseases, such as COPD and asthma [16, 17]; similarly, extracellular mtDNA has been implicated in COPD progression [18, 19]. Mitochondria represent the primary source of ROS capable of inducing the generation of cell-free DNA, including both dsDNA and mtDNA [9]. Although cigarette smoke is known to drive COPD development and alter macrophage polarization [20–22], it remains unclear how dsDNA release triggered by cigarette smoke extract (CSE) contributes to macrophage dysfunction and its associated lung inflammatory cascades, such as CSE-induced COPD pathogenesis.

In response to inflammation, macrophage polarization and the subsequent release of cytokines are critical events in the immune system’s defense against noxious insults [23]. The present study indicates that the cGAS/STING/TBK1 pathway, alongside downstream NF-κB and IRFs signaling networks, mediates dsDNA-enhanced mtROS to regulate cigarette smoke-induced lung inflammation. It achieves this by modulating M1 macrophages polarization and the release of type I cytokines and interferons. Consequently, targeting the cGAS/STING/TBK1 axis and/or dsDNA may offer novel therapeutic strategies for environmental particle-induced inflammatory lung diseases. Furthermore, activated cGAS/STING/TBK1 signaling components, excess mtROS and dsDNA/mtDNA could serve as early-stage diagnostic biomarkers for COPD.

## Materials and methods

### Patients samples collection

A total of 13 patients with chronic obstructive pulmonary disease (COPD) and 16 healthy controls (patients with non-COPD related diseases) were recruited from the Affiliated Hospital of Southwest Medical University in Luzhou, China. This study involving human samples was conducted in strict accordance with the World Medical Association declaration of Helsinki. Prior to participation, all individuals provided written informed consent authorizing the use of their blood samples for research purposes. From each participant, a 3mL sample of fresh peripheral blood was collected into a heparinized tube (BIOFIVEN, China, HH01092). The study protocol was approved by the Institutional Ethics Committee of the Southwest Medical University (Approval number: KY2024104).

### Human PBMC processing

Peripheral blood samples (3mL per patient) were collected into heparinized tube as part of standard clinical procedure. Peripheral blood mononuclear cells PBMCs were isolation via density gradient centrifugation using Ficoll (Solarbio, China, P9010). In brief, the 3 mL blood sample was diluted 1:1 (v/v) with PBS containing 2% FBS. The diluted blood was then carefully layered over 3 mL of Ficoll and centrifuged at 800 g for 30 min at room temperature. Following centrifugation, the plasma upper layer and the PBMC interphase were separately collected. The isolated PBMCs were used to analyze the STING, p-TBK1, p-NF-κB and p-IRF3 signaling pathways, while the plasma was reserved for quantifying dsDNA level.

### Measurement of double-stranded DNA in human plasma

The concentration of double-stranded DNA (dsDNA) was quantified using Quant-iTPicoGreen dsDNA assay kit (Invitrogen, USA, #2313058). Isolated human plasma, mouse BALF, and cell culture supernatants in the phenol red-free, high-glucose DMEM (ThermoFisher Scientific, USA, #31053028) were analyzed to determine dsDNA levels according to manufacturer’s instructions.

### Experimental animals and transgenic mice

All mice were housed under specific pathogen-free conditions with a regular 12-hour light/dark cycle at the Animal Experimental Center of Southwest Medical University, Luzhou, China. The 6–8 weeks old wild-type C57BL/6J and BALB/c, as well as *Lyz2*^*cre−*^*Cgas*^*fl/fl*^, *Lyz2*^*cre−*^*Tbk1*^*fl/fl*^, *Lyz2*^*cre+*^*Cgas*^*fl/fl*^, *Lyz2*^*cre+*^*Tbk1*^*fl/fl*^ and *Sting*^*−/−*^ strains, were self-bred and maintained within the facility. All animal procedures were approved by the Committee of Animal Experiments Center of Southwest Medical University and the National Institutes of Health guidelines on the care and use of animals.

### Cigarette smoke exposed mouse model

To establish the standard cigarette smoke (CS)-exposed mouse model [5], mice were exposed to CS generated from commercial cigarettes (brand name is Jiaozi, made in China, purchased from a local retailer) using a specialized exposure apparatus (HOPE-MED-8053E; Tianjin Hope Corp., China). For each exposure session, smoke from 10 cigarettes was delivered to a total of 16 mice. The mice underwent this exposure protocol twice daily for 5 consecutive days, and samples were harvested on day 6 for subsequent analyses.

For the therapeutic interventions, mice were treated via i.p. injection or i.n. instillation as follows: DNase I (5 mg/kg, i.p., Sigma, USA, D5025) was administered twice daily immediately prior to the first and following the second daily CS exposure; Mito-EMPO (5 mg/kg, i.p., MedChemExpress, USA, HY-112879) was administered twice daily, prior to each CS exposure; DNA (10 µg/mouse, i.n.) was administered twice daily, prior to each CS exposure; NAC (50 mg/kg, i.p., Sigma, USA, A9165) was administered once daily, prior to the first time CS exposure for in vitro experiments. Total DNA was extracted and purified from CSE treated RAW264.7 cells using a commercial DNA extraction kit (DC102-01, Vazyme, China) according to the manufacturer’s instructions.T

### Flow cytometry analysis

Flow cytometry was performed according to previously described protocols with minor modifications [24, 25]. Briefly, lung tissues from each group were digested in a solution containing collagenase IV (1 mg/mL; Gibco, USA, cat#17104019) and DNase I (5 mg/mL; Sigma, USA, cat#D5025) on a shaker for 30 min at 37 °C. The digested tissues were gently passed through a 200-mesh filter to obtain single-cell suspensions. Following centrifugation at 1200 rpm 5 min at 4 °C, cells were re-suspended in 100 µL FACS buffer (containing1% serum) and stained with specific antibodies. Finally, the cells were brought to a final volume of 200 µL in FACS buffer for flow cytometry analysis (NovoCyte; ACEA Biosciences). Details of the antibodies used are provided in Table 1.

**Table 1:** Antibody or Kit information

	Antibody	Source Cat#	Clone
Flow cytometry	STING-Alexa Fluor 647	Cell Signaling #90,173	E9 × 7 F
	Phospho-TBK1/NAK (Ser172)-Alexa Fluor 647	Cell Signaling #14,590	D52C2
	Phospho-NF-κB p65 (Ser536)-Alexa Fluor 647	Cell Signaling #4887	93H1
	Phospho-IRF-3 (Ser396)-Alexa Fluor 647	Cell Signaling #10,327	D6O1M
	CD86-PE	Biolegend #105,008	GL-1
	CD86-APC	Biolegend #105,012	GL-1
	CD86-Super Bright 436	eBioscience #62-0862-82	GL-1
	CD80-PE	Biolegend #104,708	16-10A1
	CD80-APC	Biolegend #104,714	16-10A1
	CD206-PE	Biolegend #141,706	C068C2
	iNOS-Percp	Biolegend #696,810	W16030C
	CD45-FITC	Biolegend #103,108	30-F11
	CD45-eFluor 506	eBioscience #69-0451-82	30-F11
	F4/80-PE-Texas Red	Invitrogen #MF48017	BM8
	CD11b-PE	eBioscience #12-0112-82	M1/70
	CD11b-Percp-Cyanine5.5	eBioscience #45-0112-82	M1/70
	CD11b-PE-Texas Red	eBioscience #RM2817	M1/70.15
	Siglec-F-PE	eBioscience #12-1702-82	1RNM44N
	CD24-PE	eBioscience #12-0242-82	M1/69
	CD64-APC	eBioscience #17-0641-82	X54-5/7.1
	CD11c-FITC	eBioscience #11-0114-82	N418
	CD11c-PE-Cy7	eBioscience #25-0114-82	N418
IF	Phospho-TBK1/NAK (Ser172)	Cell Signaling #5483	D52C2
	TOM20	Abcam #ab186735	EPR15581-54
	DNA	Merck #CBL186	AC-30-10
	CD45	Cell Signaling #70,257	D3F8Q
	Ly6G	Cell Signaling #87,048	E6Z1T
	F4/80	Cell Signaling #70,076	D2S9R
	CD86	Cell Signaling #19,589	E5W6H
WB	GAPDH	Cell Signaling #2118	14C10
	Phospho-TBK1/NAK (Ser172)	Cell Signaling #5483	D52C2
	TBK1/NAK	Cell Signaling #38,066	E8I3G
	cGAS	Cell Signaling #31,659	D3O8O
	STING	Cell Signaling #13,647	D2P2F
	Phospho-IRF-3 (Ser379)	Cell Signaling #79,945	E6F7Q
	IRF3	Cell Signaling #4302	D83B9
	Phospho-NF-κB p65 (Ser536)	Cell Signaling #3033	93H1
	NF-κB	Cell Signaling #8242	D14E12
	c-Caspase3	Cell Signaling #9664	Asp175
	p-MLKL	Sigma #MABC1158	7C6.1
	MLKL	Sigma #MABC604	3H1
	GSDMD	Abcam #ab209845	EPR19828

**Table:** Flow cytometry gating strategies

Macrophage	CD45^+^CD11b^+^F4/80^+^(Fig. 3)
Macrophage CD80^+^	CD45^+^CD11b^+^F4/80^+^CD80^+^(Fig. 3)
Macrophage CD86^+^	CD45^+^CD11b^+^F4/80^+^CD86^+^(Fig. 3)
Dendritic cells CD86^+^	CD45^+^CD11b^+/-^CD11c^+^CD64^-^CD24^+^CD86^+^
Neutrophils	CD45^+^Ly6G^+^
Eosinophils	CD45^+^Ly6G^-^Siglec-F^+^CD11b^+^CD11c^-^

"+" indicates positive, "-" indicates negative

### Cell culture and treatment

The RAW 264.7 macrophage-like cell line was purchased from ATCC (United States) and maintained as previously described [24] at 37 °C with 5% CO_2_ in Dulbecco’s modified Eagle medium (DMEM; BasalMedia, China, L110KJ) supplemented with 10% fetal bovine serum (FBS, Corning Inc., United States, 35-076-CV) 50 U/mL penicillin and 50 µg/mL streptomycin.

Cigarette smoke extract (CSE) was prepared according to previously described protocols [26, 27]. Briefly, the smoke from four cigarettes was bubbled into 15 mL of DMEM and then passed through a 0.2 μm filter. The filtered solution was designated as the 100% cigarette smoke extract stock solution for the subsequent in vitro studies. The stock solution was stored at 4 ℃ and used within 24 h.

Total DNA was extracted and purified from CSE-treated RAW 264.7 cells using a commercial kit from Vazyme (China, DC102-01) and quantified using a Nanodrop 1000 spectrophotometer. Cells were then transfected with 1 µg of DNA using DOTAP (Roche, Germany, 11202375001) transfection reagent according to the manufacturer’s instructions. Following transfection, the cells were washed to remove residual reagent and exposed to CSE (1%) for 18 h prior to collection for subsequent analyses.

Cells were cultured in the presence of Ethidium bromide (EtBr, 150 ng/mL, MedChemExpress, USA, HY-D0021) for 4 days. The cells were then washed with PBS and re-culture overnight without EtBr prior to CSE exposure.

Cells were pretreated with DNase I (1 µg/mL), NAC (500 µM), Z-VAD-FMK (10 µM, MedChemExpress, USA, HY-16658B), Mito-TEMPO (2 µM) or c-di-AMP (5 µg/mL, MedChemExpress, USA, HY-12326) for 2 h prior to CSE exposure.

Lentiviral transduction: Lentiviral transduction was performed as previously described [28, 29]. Briefly, cells were transduced with lentiviral vectors express si-NC, si-cGAS (GATTGAGCTACAAGAATAT), si-STING (CGAAATAACTGCCGCCTCA), or si-TBK1 (CCACAAATTTGATAAGCAA) (GeneChem Technology, China) at a multiplicity of infection (MOI) of 20 to knock down gene expression. Transduced cells were then treated with the indicated reagents for each experiment. The si-NC group served as the negative control. GFP serves as a reporter for the lentiviral vector; thus, the GFP-positive indicates successful transfection.

To generate cGAS^−/−^, STING^−/−^ and TBK1^−/−^ knockout lines, RAW 264.7 cells were genetically modified using the CRISPR-Cas9 system. The specific gRNA target sequence utilized were as following: cGAS (g1: GGTGGCTCCTGCCGCGTCCATGG; g2: ATCCGGAGCTGCCCGCCGTACGG), STING (g1: ACACCTCAAGGAGACGCCCTGGG; g2: GAAGGCTGTGCGCGCCTGCCTGG), and TBK1 (g1: CTGCAAATGTCTTCCGAGGAAGG; g2: CCTGGCCTAGGATATCAGACAGG). Following transfection, the cells were treated with or without CSE for 18 h prior to downstream analysis.

### mtROS measurement

The mitochondrial superoxide, mainly mtROS, was detected by mitoSOX mitochondrial superoxide indicator (Invitrogen, USA, M36008) and the fluorescence was read by flow cytometry. The protocol was based on the manufacturer’s instructions.

### Determination of iNOS activity

Cellular iNOS activity was determined using a commercial nitric-oxide synthase assay kit (Beyotime Institute of Biotechnology, China, S0025) according to the manufacturer’s instructions. Following 18 h of CSE treatment, cells were harvested and resuspended in 100 µL of iNOS assay buffer. Subsequently, 100 µL of the iNOS assay reaction solution (comprising 50% iNOS assay buffer, 38.8% H_2_O, 5% L-Arginine solution, 5% 0.1 mM NADPH, 0.2% DAF-FM DA) was added to each sample, following by incubation at 37 ℃ for 30 min. After incubation, the cells were washed twice by PBS, and fluorescence intensity was quantified via flow cytometry.

### Apoptosis analysis

RAW264.7 cells were seeded into 6-well plate and assigned to the treatment groups specified for each experiment. Following the designated treatment, cells were harvested, and approximately 1 × 10^6^ cells were collected per sample. The cells were then resuspended in 100 µL of binding buffer (5 µL Annexin V-FITC and 5 µL Propidium iodide (PI), Vazyme, China, A213-10), followed by a 10 min incubation at room temperature in the dark. Apoptotic cells were quantified using flow cytometry, and the resulting data were analyzed using FlowJo 10 software.

### Western blot

Western blotting was performed as previous described [24, 29]. Lung tissues and cells were homogenized with 5 mm steel beads using a tissue homogenizer, then solubilized in ice-cold radio immunoprecipitation assay (RIPA) buffer supplemented with a protease inhibitor cocktail. Proteins were separated via 12% SDS-PAGE and transferred onto microporous polyvinylidene difluoride (PVDF) membrane at 200 mA for 2 h using a wet transfer method. After blocking with 5% nonfat milk in Tris-buffered saline/Tween for 1 h at room temperature, membranes were then incubated with primary antibodies (Table 1) overnight at 4 °C. The membranes were then incubated with horseradish peroxidase (HRP)-conjugated anti-rabbit or anti-mouse IgG secondary antibodies. Protein bands were visualized using an ECL Western Blotting kit (4 A Biotech, China, 4AW011-20), and chemiluminescent signals were captured using a BioRad ChemiDoc Touch system.

### Florence immunostaining

Immunofluorescence staining was performed according to standard laboratory protocols. Briefly, cells cultured on chamber slides were fixed with 4% formaldehyde and permeabilized with 0.3% Triton X-100 in PBS for 10 min at room temperature (around 20 °C). After blocking with 1% bovine serum albumin (BSA), the specimens were incubated overnight at 4 °C with primary antibodies against TOM20, DNA, p-TBK1. Cells were then stained with Alexa Fluor 555- or Alexa Fluor 488-conjugated secondary antibody (1:500; Invitrogen, United States, cat. no. A32727 and A28175). Nuclei were counterstained using DAPI (Beyotime Institute of Biotechnology, China, C1002). Images were acquired from at least six random fields per sample using a confocal microscopy (Olympus, Japan, SpinSR10). The mean fluorescence intensity was quantified using ImageJ software.

### Multiplex Immunohistochemistry (mIHC)

Formalin-fixed, paraffin-embedded (FFPE) whole tissue Sections (4 μm thick) were subjected to multiplexed immunohistochemistry (mIHC) utilizing a sequential primary antibody staining approach coupled with a TSA 7-color kit (abs50015-100T, Absinbio, Shanghai), followed by counterstaining with DAPI. Briefly, deparaffinized tissue slides were incubated with a primary antibody for 30 min, followed by a 10 min incubation with an anti-mouse horseradish peroxidase (HRP)-conjugated secondary antibody (abs50015-02, Absinbio, Shanghai). The fluorescent signal was subsequently developed for precisely 10 min using TSA 520 according to the manufacturer’s protocol. Following a wash in TBST buffer, the sections were transferred to a preheated citrate solution (90 °C) and subjected to microwave-mediated antigen retrieval at 20% maximum power for 15 min. The slides were then allowed to cool to room temperature within the same solution. Intermittent washes with Tris buffer were performed between all experimental steps. This sequential stripping and labeling procedure was repeated in order for the remaining antibody/fluorophore pairs (each color paired antibody as shown in the result figures). Finally, nuclei were stained by applying 2 drops of DAPI (abs47047616, Absinbio, Shanghai), washed with distilled water, and manually mounted with coverslips. After air-drying, slides were imaged using a Pannoramic MIDI II digital slide scanner (3DHISTECH), and subsequent image analysis was executed via the Indica Halo software platform.

### Total mRNA extraction

The real-time qPCR sample preparation was performed according to established laboratory protocols [28]. Following cell stimulation, total RNA was extracted using an RNA extraction kit (Vazyme, China, cat#RC112-01). Subsequently, the full-length cDNA was synthesized using a reverse transcription kit (Vazyme, China, cat#R323-01). The resulting cDNA products were stored in the freezer (at -20 °C) for downstream analysis.

### Real-time qPCR

RT-qPCR was performed on a LightCycler 480 Ⅱ instrument (Roche, Switzerland) using a commercial kit (Vazyme, China, cat#Q711-02) according to the manufacturer’s instructions and a previously described protocol [28]. The amplification consisted of an initial denaturation at 95 °C for 10s, followed by 40 cycles of 95 °C for 10s, 58 °C for 10s, and 72 °C for 10s; with a final elongation step at 65 °C for 1 min. Relative gene expression levels were calculated using the 2^−ΔΔCt^ method, with GAPDH serving as the internal control. Primer sequences are listed in Table 2.

### Quantification of mtDNA release by qPCR

The RAW264.7 cells (2 × 10^6^) were harvested and resuspended in 170 µL of digitonin buffer (150 mM NaCl, 50 mM HEPES pH 7.4, and 25 µg/mL digitonin (MedChemExpress, United States, HY-N4000)). The cell suspension was incubated on a rotator at room temperature for 10 min, followed by centrifugation at 16,000×g for 25 min at 4 °C. To determine total mtDNA, the resulting pellets were resuspended in 340 µL of lysis buffer (containing 5 mM EDTA and proteinase K (Vazyme)) and digested overnight at 55 °C. Following digestion, samples were diluted (1:20 to 1:100) with ddH_2_O and incubated at 95 °C for 20 min to inactivate proteinase K prior to qPCR analysis. To measure cytosolic mtDNA (cmtDNA), the supernatant was collected and diluted 1:20, qPCR was performed using specific primers (Table 2). The abundance of cmtDNA in the supernatant was normalized against the total mtDNA obtained from the corresponding cell pellet.

**Table 2:** Primer sequences of Real-time qPCR

Primer	Sequence (5’-3’)
Gapdh	F: ATGCCAGTGAGCTTCCCGTTCAG R: CATCACTGCCACCCAGAAGACTG
Il-1b	F: TGGACCTTCCAGGATGAGGACA R: GTTCATCTCGGAGCCTGTAGTG
Il-6	F: ACCGCTATGAAGTTCCTCTCTGCAA R: AAGCCTCCGACTTGTGAAGTGGT
Tnf-α	F: GGTGCCTATGTCTCAGCCTCTT R: GCCATAGAACTGATGAGAGGGAG
Ifna	F: GGATGTGACCTTCCTCAGACTC R: ACCTTCTCCTGCGGGAATCCAA
Ifnb	F: GCCTTTGCCATCCAAGAGATGC R: ACACTGTCTGCTGGTGGAGTTC
D-loop1	F: AATCTACCATCCTCCGTGAAACC R: TCAGTTTAGCTACCCCCAAGTTTAA
D-loop2	F: CCCTTCCCCATTTGGTCT R: TGGTTTCACGGAGGATGG
D-loop3	F: TCCTCCGTGAAACCAACAA R: AGCGAGAAGAGGGGCATT
mt-16s	F: CACTGCCTGCCCAGTGA R: ATACCGCGGCCGTTAAA
mt-Nd4	F: AACGGATCCACAGCCGTA R: AGTCCTCGGGCCATGATT

### ELISA

Lung homogenates were utilized for cytokines analysis. The ELISA protocol followed the manufacturer’s instructions and established methodologies [24, 30]. The following ELISA kits were purchased from Shanghai Jianglai Industrial Limited by Share Ltd., Shanghai, China: IL-1β (JL18442), IL-6 (JL20268), TNF-α (JL10484), IFN-α (JL12034), IFN-β (JL20219) and IL-2 (JL20256).

### Statistical analysis

Data analysis was performed using SPSS 17.0. All data are shown as mean ± standard deviation (s.d.). Differences between two groups were evaluated using the Student’s t-test. For comparisons among multiple groups, a One-way analysis of variance (one-way ANOVA) followed by Tukey’s multiple comparisons test was applied. A *P* or adjusted *p* value of less than 0.05 was considered statistically significant.

## Results

### Activation of STING/TBK1 signaling in COPD patients

**Fig. 1:**
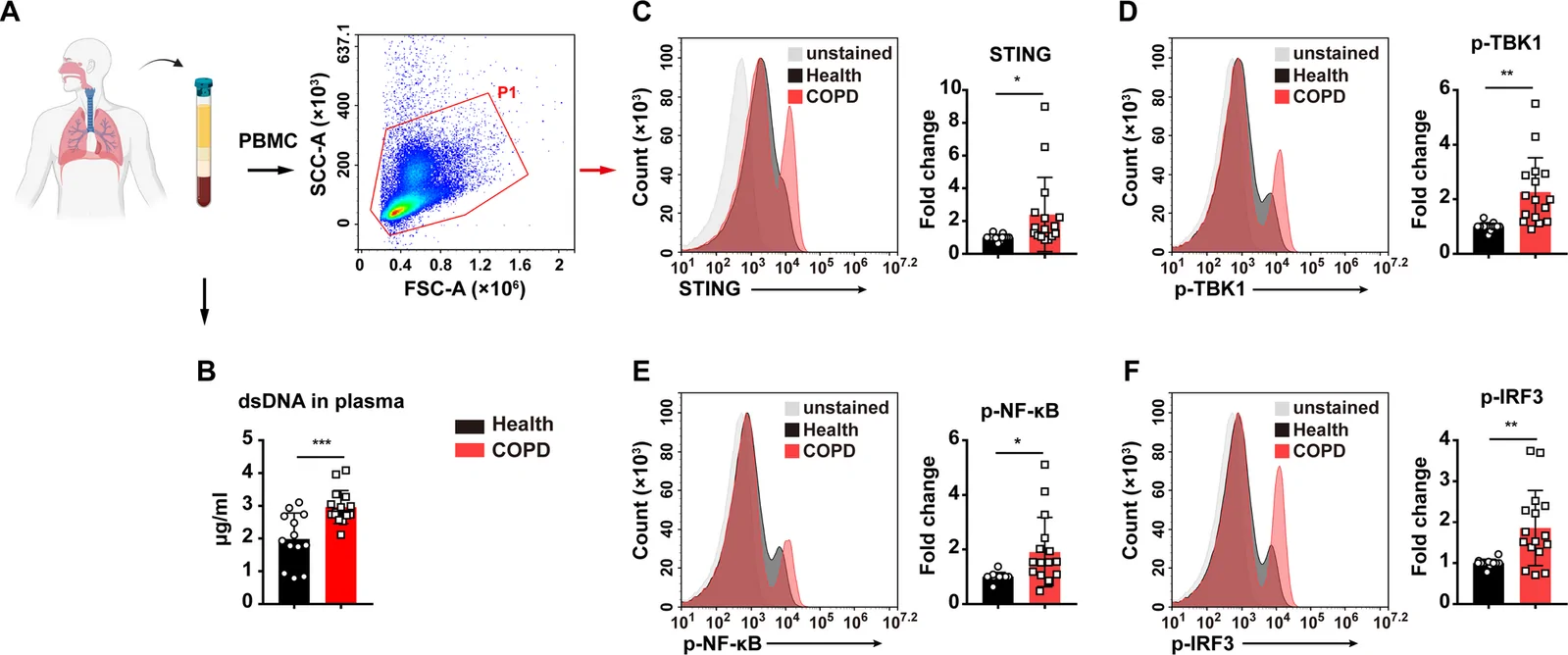
COPD patients exhibit elevated dsDNA levels and activated STING/TBK1 signaling. (**A**): Schematic workflow illustrating the collection of blood samples from patients with COPD (*n* = 16) and healthy donors (*n* = 13) for the isolation of PBMCs and plasma. (**B**): Quantification of plasma dsDNA concentration. (C-F) Flow cytometry analysis of human PBMCs showing expression levels of STING (**C**), as well as phosphorylated TBK1 (p-TBK1) (**D**), phosphorylated NF-κB (p-NF-κB) (E) and phosphorylated IRF-3 (p-IRF-3) (**F**). Data are presented as mean ± SD and were analyzed using a two-tailed Student’s t-test. *p < 0.05, **p < 0.01

The release of self-dsDNA has been closely linked to the pathogenesis of COPD [31], with elevated serum dsDNA levels serving as a significant risk factor that negatively impacted patient survival rates [32]. The cGAS/STING signaling network plays a critical role in the development and progression of lung disease [9, 33]. To evaluate dsDNA production and the status of STING-related signaling in a clinical cohort, blood samples were collected from 16 COPD patients and 13 healthy donors (none-COPD patients’ controls) to isolate PBMCs for downstream analysis (Fig. 1A). Our results confirmed that plasma dsDNA levels were significantly elevated in COPD patients compared to controls (Fig. 1B). Consistently, flow cytometry analysis of the clinical PBMC samples revealed that total STING (Fig. 1C), alongside the phosphorylation of downstream effectors-namely p-TBK1 (Fig. 1D), p-NF-κB (Fig. 1E) and p-IRF3 (Fig. 1F) was markedly increased in the COPD group. Taken together, these findings demonstrate that excessive self-DNA release and the subsequent activation of the STING/TBK1 signaling pathway are key characteristics of COPD pathogenesis.

### The cGAS/STING/TBK1 signaling is associated with CSE-induced M1 macrophages polarization in vitro

Lung macrophages serve as a primary defense mechanism against inhaled particle-induced inflammatory responses. Therefore, characterizing macrophage phenotypes during cigarette smoke extract (CSE) stimulation is of critical importance. Previous studies have demonstrated that NF-κB/IRF3 signaling is strongly linked to M1 macrophage reprogramming [34, 35]. Given that elevated STING/TBK1 signaling has been observed in COPD patients, we investigated whether STING-associated signaling factors influence p-TBK1 activation and regulate macrophage polarization under CSE exposure in vitro.

**Fig. 2:**
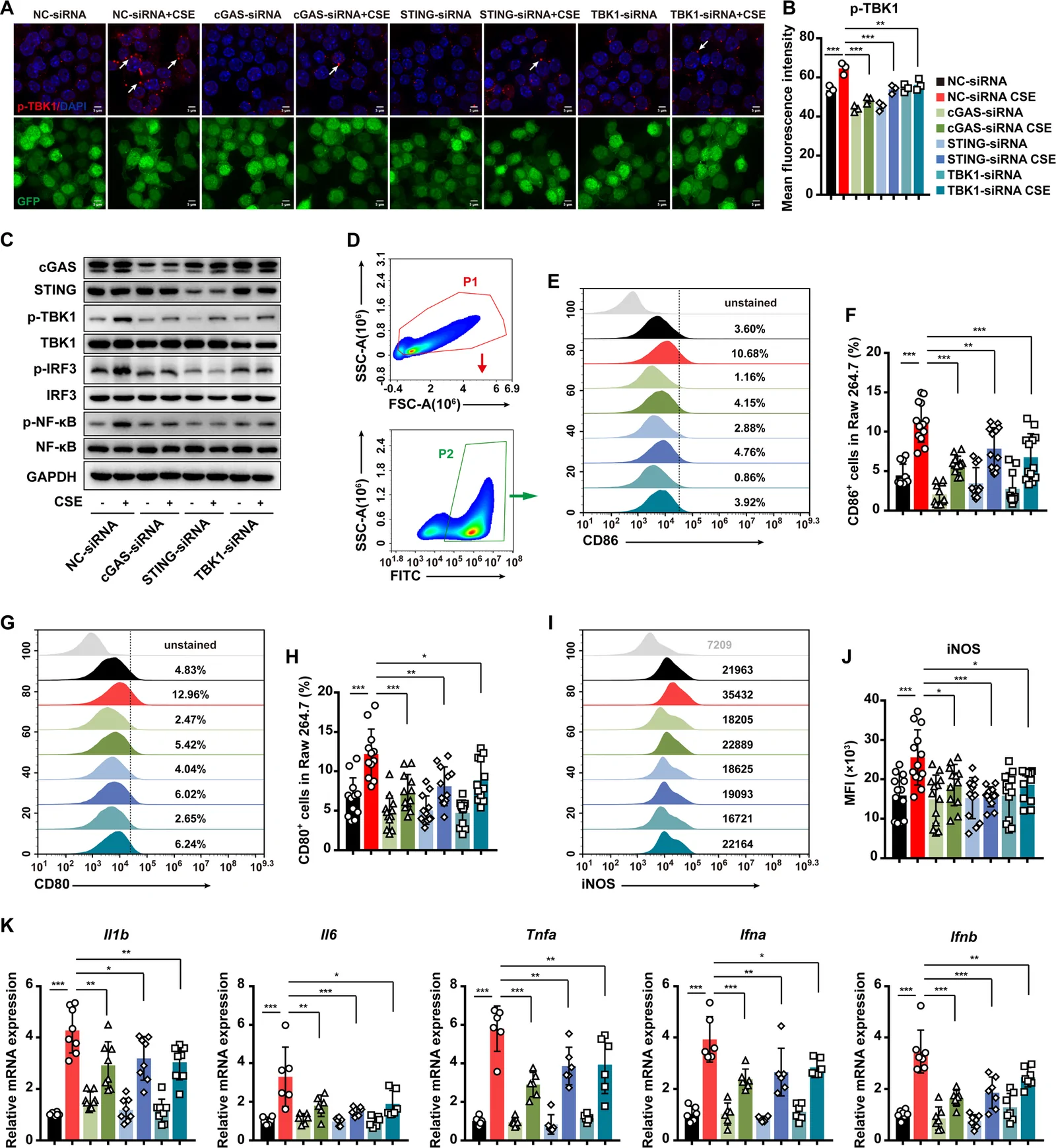
Cigarette smoke extract (CSE) alerts inflammatory cytokine expression and cGAS/STING/TBK1 signaling in macrophage-like RAW 264.7 cells. (**A**-**B**): Immunofluorescence staining of phosphorylated TBK1 (p-TBK1) following siRNA-mediated knockdown of cGAS/STING/TBK1 and exposure to CSE (*n* = 3 in each group). (**C**): Western blot analysis of NF-κB, IRF3, cGAS, STING and TBK1 signaling following cGAS/STING/TBK1 siRNA knockdown (*n* = 3 in each group). (**D**): Flow cytometry gating strategies and quantitative analysis of CD86 (**E**-**F**), CD80 (**G**-**H**) and iNOS (**I**-**J**) expression (*n* = 11–13 per group). (**K**): Relative mRNA expression of Il1b, Il6, Tnf-α, Ifn-α and Ifn-β determined by qPCR (*n* = 6–8 per group). Data are presented as mean ± SD; * indicates *p* < 0.05, ** indicates *p* < 0.01, *** indicates *p* < 0.001

The macrophage-like cell line RAW 264.7 is a well-established model for studying macrophage function. In this study, we utilized these cells to investigate the underlying molecular mechanisms of CSE-induced responses. RAW264.7 cells were transfected with siRNAs targeting cGAS, STING, or TBK1 (cGAS-siRNA, STING-siRNA, and TBK1-siRNA) in the presence or absence of CSE co-stimulation; negative control siRNA (NC-siRNA) served as the experimental control. Immunofluorescent analysis revealed that CSE treatment alone dramatically enhanced TBK1 phosphorylation (p-TBK1). Conversely, the knockdown of cGAS, STING and TBK1 significantly decreased p-TBK1 levels (Fig. 2A-B). Subsequent western blot analysis confirmed these findings (Fig. 2C) and demonstrated that CSE-mediated activation of downstream transcription factors p-NF-κB and p-IRF3 was dependent on cGAS, STING and TBK1.

Furthermore, flow cytometry analysis (Fig. 2D) demonstrated an increase in M1 macrophage following CSE treatment, as evidenced by expanded populations of CD86^+^ (Fig. 2E-F) and CD80^+^ (Fig. 2G-H) RAW264.7 cells, alongside an elevated mean fluorescence intensity (MFI) of iNOS (Fig. 2I-J). However, these phenotypic changes were markedly attenuated upon inhibition of the cGAS/STING/TBK1 signaling pathway, indicating that CSE drives M1 macrophage polarization via this axis. The M1 response was further substantiated by the upregulation of pro-inflammatory cytokines (*Il-1b*, *Il6* and *Tnfa*) and interferons (*Ifna* and *Ifnb*) at the mRNA level. Consistent with our protein-level data, this CSE-induced M1 reprogramming was found to be dependent on cGAS, STING and TBK1 signaling (Fig. 2K).

To definitively confirm the role of STING in CSE-induced pathogenesis, we utilized the STING activator cyclic di-AMP (c-di-AMP). Both CSE and c-di-AMP independently enhanced the CD80^+^ and CD86^+^ populations in RAW 264.7 cells. Moreover, combined treatment with c-di-AMP and CSE further synergistically promoted CD80 and CD86 expression compared to either treatment alone (Figure S1A-S1B).

Collectively, these results reveal that CSE triggers macrophage M1 polarization through the activation of the cGAS/STING/TBK1/NF-κB/IRF3 signaling network.

### The cGAS/STING/TBK1 signaling pathway regulates CS-triggered M1 immune response in vivo

Because RAW 264.7 is a BALB/c-derived macrophage-like cell line, we next sought to validate our findings in vivo. BALB/c mice were exposed to cigarette smoke (CS) twice daily for up to 5 days (Figure S3A), after which lung macrophages were isolated and analyzed (CD45^+^CD11b^+^F4/80^+^) (Figure S3B). CS exposure led to a significant increase in both absolute number and percentage of CD80^+^ (Figure S3C) and CD86^+^ (Figure S3D) macrophages per lung, alongside an elevated mean fluorescence intensity of iNOS (Figure S3F). Conversely, while the absolute number of CD206^+^ (Figure S3E) macrophage increased significantly but their percentage remained unchanged compared to the control group. Taken together, these results demonstrate that acute CS exposure selectively drives an M1, rather than an M2, macrophage polarization response in vivo.

**Fig. 3:**
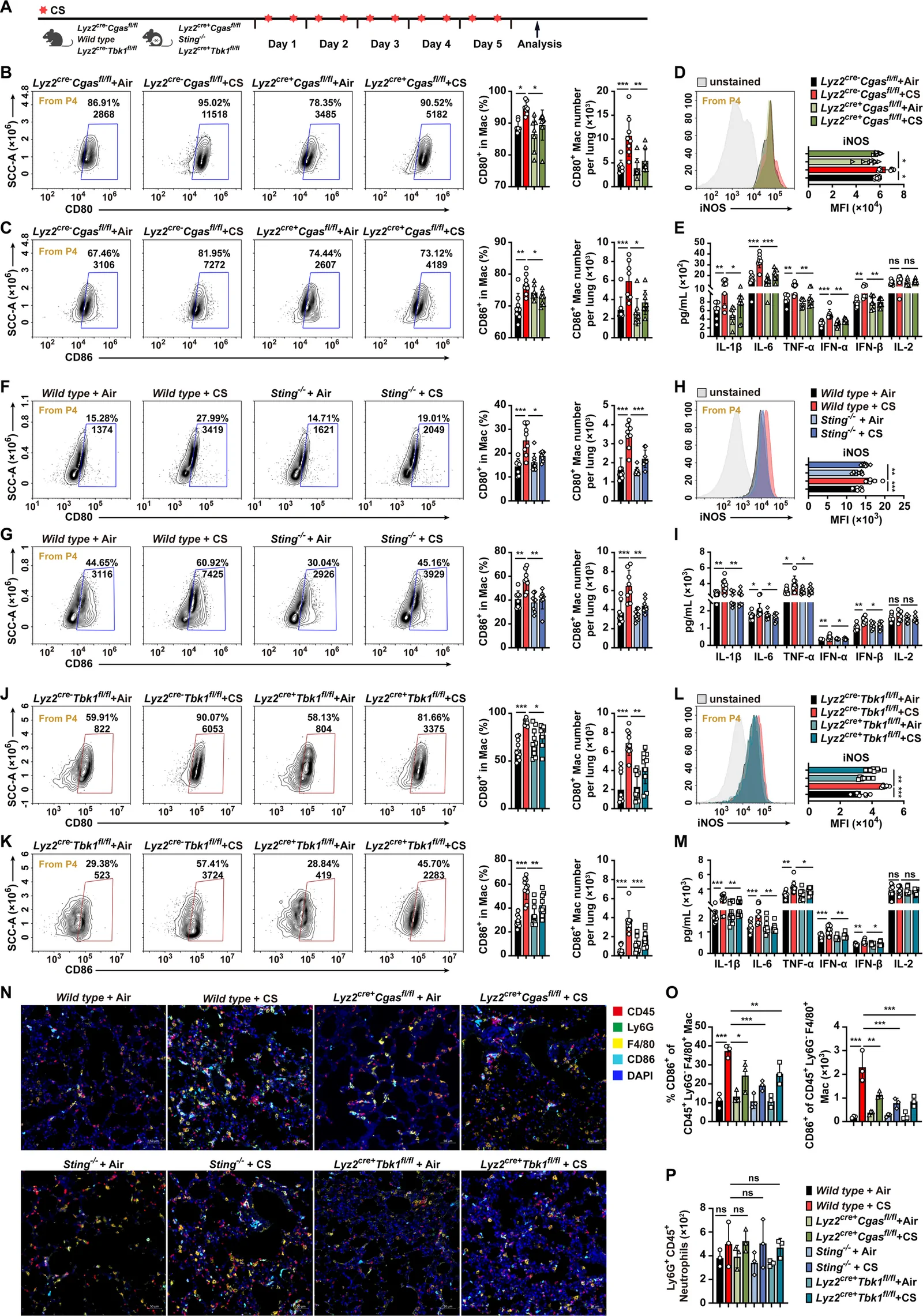
Effects of Cigarette smoke (CS) on lung macrophage M1 polarization and inflammatory cytokine expression via the cGAS/STING/TBK1 pathway. (**A**): Schematic overview of the experimental design: wild type, *Lyz2*^*cre−*^*Cgas*^fl/fl^, *Lyz2*^*cre−*^*Tbk1*^fl/fl^, *Lyz2*^*cre+*^*Cgas*^fl/fl^, *Lyz2*^*cre+*^*Tbk1*^fl/fl^ and *Sting*^−/−^ mice were exposed to CS twice daily for five days, with lungs tissues harvested for analysis on day 6 (*n* = 7–11 per group). (**B**): Total cell number and percentage of CD80^+^ and (**C**) CD86^+^ macrophages in the lungs of *Lyz2*^*cre−*^*Cgas*^fl/fl^ and *Lyz2*^*cre+*^*Cgas*^fl/fl^ mice following CS exposure. (**D**): Mean fluorescence intensity (MFI) of iNOS expression in lung macrophages from *Lyz2*^*cre−*^*Cgas*^fl/fl^ and *Lyz2*^*cre+*^*Cgas*^fl/fl^ mice measured by flow cytometry. (**E**): Cytokine levels (IL-1β, IL-6, TNF-α, IFN-α, IFN-β and IL-2) in lung tissues of *Lyz2*^*cre−*^*Cgas*^fl/fl^ and *Lyz2*^*cre+*^*Cgas*^fl/fl^ mice. Total cell number and percentage of (**F**) CD80^+^ and (**G**) CD86^+^ lung macrophages in wild type C57BL/6J and *Sting*^−/−^ mice following CS exposure. (**H**): Mean fluorescence intensity of iNOS expression in lung macrophages from wild type C57BL/6J and *Sting*^−/−^ mice. (**I**): Cytokine levels in lung tissues of wild type C57BL/6J and *Sting*^−/−^ mice. Total cell number and percentage of (**J**) CD80^+^ and (**K**) CD86^+^ lung macrophages in *Lyz2*^*cre−*^*Tbk1*^fl/fl^ and *Lyz2*^*cre+*^*Tbk1*^fl/fl^ mice following CS exposure. (**L**): Mean fluorescence intensity of iNOS expression in lung macrophages from *Lyz2*^*cre−*^*Tbk1*^fl/fl^ and *Lyz2*^*cre+*^*Tbk1*^fl/fl^ mice. (**M**): Cytokine levels in lung tissues of *Lyz2*^*cre−*^*Tbk1*^fl/fl^ and *Lyz2*^*cre+*^*Tbk1*^fl/fl^ mice. (**N**): Multiplexed immunohistochemistry staining M1 macrophage and neutrophil in wild type, *Lyz2*^*cre−*^*Cgas*^fl/fl^, *Lyz2*^*cre−*^*Tbk1*^fl/fl^, *Lyz2*^*cre+*^*Cgas*^fl/fl^, *Lyz2*^*cre+*^*Tbk1*^fl/fl^ and *Sting*^−/−^ mice exposed to CSE. (**O**) Percentage and absolute count of CD86^+^ macrophage. (**P**) Absolute count of neutrophils. Data are presented as mean ± SD; * indicates *p* < 0.05, ** indicates *p* < 0.01, *** indicates *p* < 0.001

Here, we further investigated whether the cGAS/STING/TBK1 axis contributes to cigarette smoke (CS)-induced pulmonary inflammation and macrophage polarization in vivo. To explore this CS-induced immune response, we utilized genetically modified mice, including macrophage-specific deletions (*Lyz2*^*cre−*^*Cgas*^*fl/fl*^, *Lyz2*^*cre−*^*Tbk1*^*fl/fl*^, *Lyz2*^*cre+*^*Cgas*^*fl/fl*^, *Lyz2*^*cre+*^*Tbk1*^*fl/fl*^) and global STING knockouts (*Sting*^*−/−*^) (Fig. 3A and Figure S4A). Mice were exposed to CS twice daily for up to 5 days, after which lung tissues were harvested to analyze the macrophages response using the indicated FACS gating strategies (Figure S2 and Figure S4B).

Compared to air-exposed controls, CS exposure significantly increased both the percentage and total cell number of M1 macrophages. Specifically, the percentages and absolute numbers of CD80^+^ (Fig. 3B, F and J) and CD86^+^ cells (Fig. 3C, G and K) were elevated in CS-exposed mice. Furthermore, the mean fluorescence intensity (MFI) of iNOS was markedly upregulated (Fig. 3D, H and L). Notably, these CS-triggered M1 immune responses were strictly dependent on cGAS/STING/TBK1 signaling. In contrast to control mice, myeloid-specific cGAS-deficient mice (*Lyz2*^*cre+*^*Cgas*^*fl/fl*^) displayed no significant CS-associated changes in M1 macrophage markers (Fig. 3B-D). Consistent with these findings, the CS-induced M1 response was largely abolished in mice lacking either STING (*Sting*^*−/−*^) (Fig. 3F-H) or TBK1 (*Lyz2*^*cre+*^*Tbk1*^*fl/fl*^) (Fig. 3J-K).

Additionally, CS exposure significantly upregulated pro-inflammatory cytokines (IL-1β, IL-6, TNF-α, but not IL-2) as well as type I interferon (IFN-α, and IFN-β) (Fig. 3E, I and M). This induction was heavily reliant on cGAS/STING/TBK1 signaling; the CS-mediated overexpression of these factors was dramatically blunted in each of the deficient group (*Lyz2*^*cre+*^*Cgas*^*fl/fl*^, *Sting*^*−/−*^, and *Lyz2*^*cre+*^*Tbk1*^*fl/fl*^). These findings were further validated by multiplexed immunohistochemistry and subsequent quantification of CD86^+^ cells, evaluating both percentages and absolute cell counts (Fig. 3N-O). Conversely, no such changes were observed regarding CD206 expression and neutrophil levels (Figure S4 and Fig. 3P). Moreover, the lack of changes in eosinophils, neutrophils, and CD86^+^ dendritic cells (DCs) were verified in vivo which were further confirmed in genetic modified mice (Figure S5 and Figure S6).

Taken together, these findings demonstrate that CS-induced in vivo macrophages M1 polarization and the associated inflammatory response are mediated potentially via the activation of the cGAS/STING/TBK1 signaling pathway.

### dsDNA release mediates CSE-induced M1 macrophages polarization

**Fig. 4:**
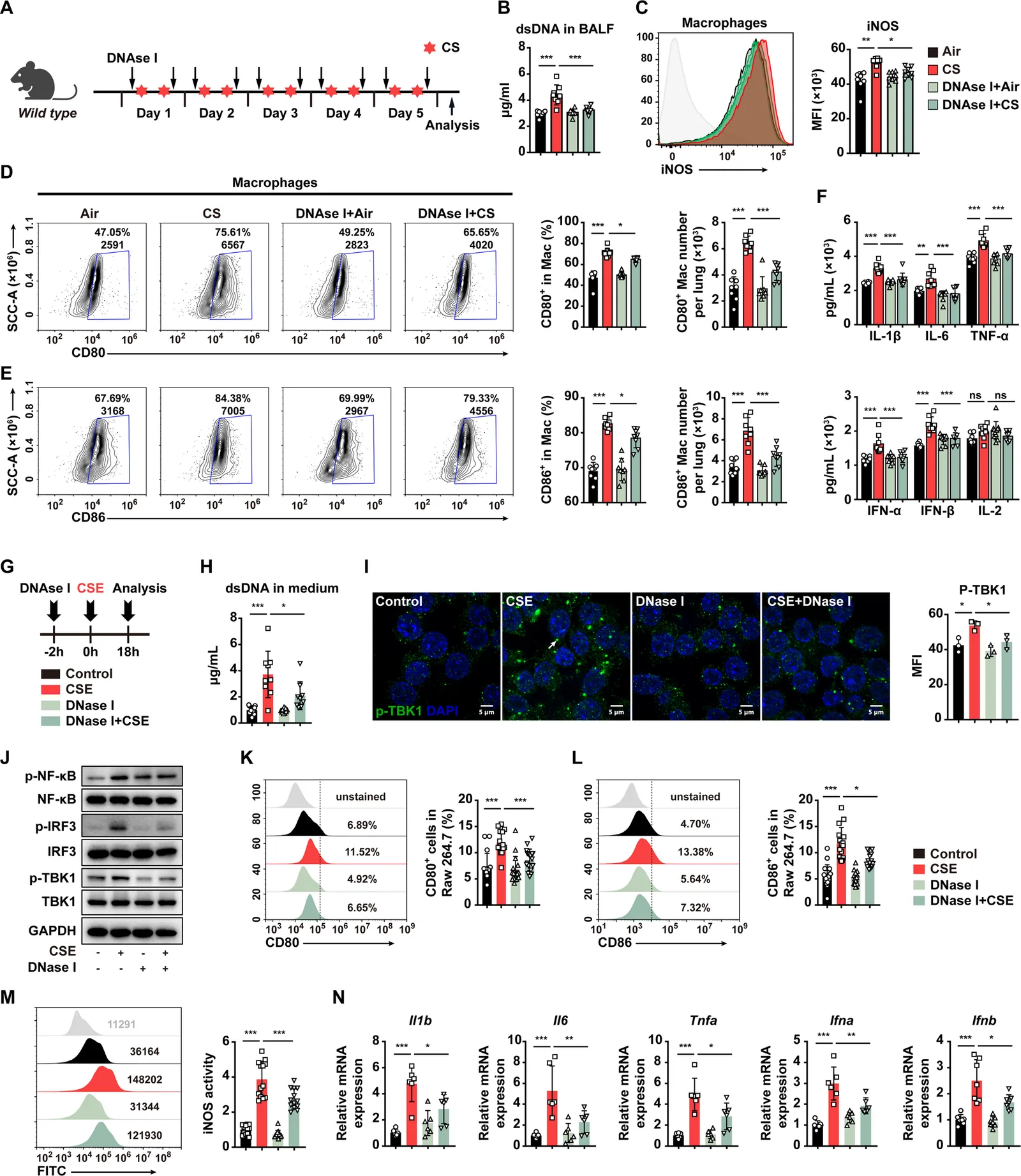
DNase I attenuates CSE-induced inflammatory responses by inactivating cGAS/STING/TBK1 pathway

As previously described, dsDNA is involved in COPD pathogenesis and is highly correlated with patient survival rates [31, 32]. ‘Mislocalized’ self-DNA can be sensed by cGAS/STING signaling, contributing to the development of inflammatory diseases [36, 37]. Our results have indicated that CS promotes M1 macrophage polarization via cGAS/STING/TBK1 signaling networks. Therefore, we sought to verify whether CS exposure drives M1 macrophage programming specifically by promoting dsDNA release. To test this, in addition to establishing the CS-induced mouse model, mice were administrated DNase I twice daily both before and after CS exposure for up to 5 days (Fig. 4A).

We found that CS significantly elevated dsDNA levels in mouse broncho alveolar lavage fluid (BALF), an effect that was marked by a sharp decline following DNase I treatment (Fig. 4B). Consistently, we demonstrated that DNase I administration blocked M1 macrophage polarization. This was evidenced by the downregulated activity of iNOS (Fig. 4C), decreased percentages of CD80^+^ (Fig. 4D) and CD86^+^ (Fig. 4E) cells, and a reduction in pro-inflammatory cytokines (IL-1β, IL-6, TNF-α, IFN-α, and IFN-β) (Fig. 4F) that were otherwise elevated by CS, with the expectation of IL-2. In addition, these findings were further validated by multiplexed immunohistochemistry and subsequent quantification of CD86^+^ cells, evaluating both percentages and absolute cell counts (Figure S7). Taken together, these results confirm that dsDNA release is critically associated with CS-induced M1 macrophage reprogramming and subsequent cytokine release.

To further validate these in vivo findings, RAW 264.7 cells were pretreated with DNase I for 2 h prior to CSE exposure (Fig. 4G). Extracellular dsDNA levels in the cell culture medium, which were elevated following CSE stimulation compared to the control group, were significantly reduced by DNase I co-treatment (Fig. 4H). Furthermore, DNase I administration attenuated the CSE-induced phosphorylation of TBK1 (p-TBK1), IRF3 (p-IRF3) and NF-κB (p-NF-κB)(Fig. 4I-J). Consistently, the CSE-induced increases in the percentages of CD80^+^ (Fig. 4K) and CD86^+^ (Fig. 4L) macrophages, the secretion of pro-inflammatory cytokines (*Il1b, Il6, Tnfa, Ifna* and *Ifnb*) (Fig. 4N), and iNOS activity (Fig. 4M) were all significantly blunted by DNase I treatment.

Conversely, to complement the DNase I experiments, RAW 264.7 cells were stimulated with exogenous dsDNA 2 h prior to CSE administration (Figure S8A). Combined treatment with dsDNA and CSE further enhanced the activation of TBK1, IRF3 and NF-κB compared to CSE treatment alone (Figure S8B). This was accompanied by a marked promotion of M1 macrophages polarization, as evidenced by increased expression of CD80 (Figure S8C), CD86 (Figure S8D), and pro-inflammatory cytokines (*Il1b, Il6, Tnfa, Ifna* and *Ifnb*) (Figure S8F), alongside elevated cellular iNOS activity (Figure S8E).

To investigate the underlying mechanism of dsDNA in vivo, the mice were administered dsDNA prior to each CS exposure (Figure S9A). In-depth analysis of lung macrophages revealed that CS exposure shifted cells toward an M1 phenotype, significantly upregulating both the percentages and absolute numbers of CD80^+^ (Figure S9B) and CD86^+^ (Figure S9C) cells, the levels of pro-inflammatroy cytokines (IL-1β, IL-6, TNF-α, IFN-α, and IFN-β, but not IL-2) (Figure S9E), and the MFI of iNOS (Figure S9D). Notably, this M1 polarization was further exacerbated in the dsDNA plus CS group compared to the CS-alone group. Taken together, these findings demonstrate that CSE/CS-induced dsDNA release drives M1 macrophage polarization both in vivo and in vitro.

### CSE-induced apoptosis but not pyroptosis or necroptosis, promotes dsDNA release

CSE is well known to induce ROS generation, which is highly associated with DNA damage and subsequent dsDNA release [9, 38]. Furthermore, ROS generation has been implicated in macrophage reprogramming [39]. In this study, we observed that both ROS (Figure S2G) and particularly mtROS (Figure S2H) were dramatically overproduced in macrophages following CS exposure.

**Fig. 5:**
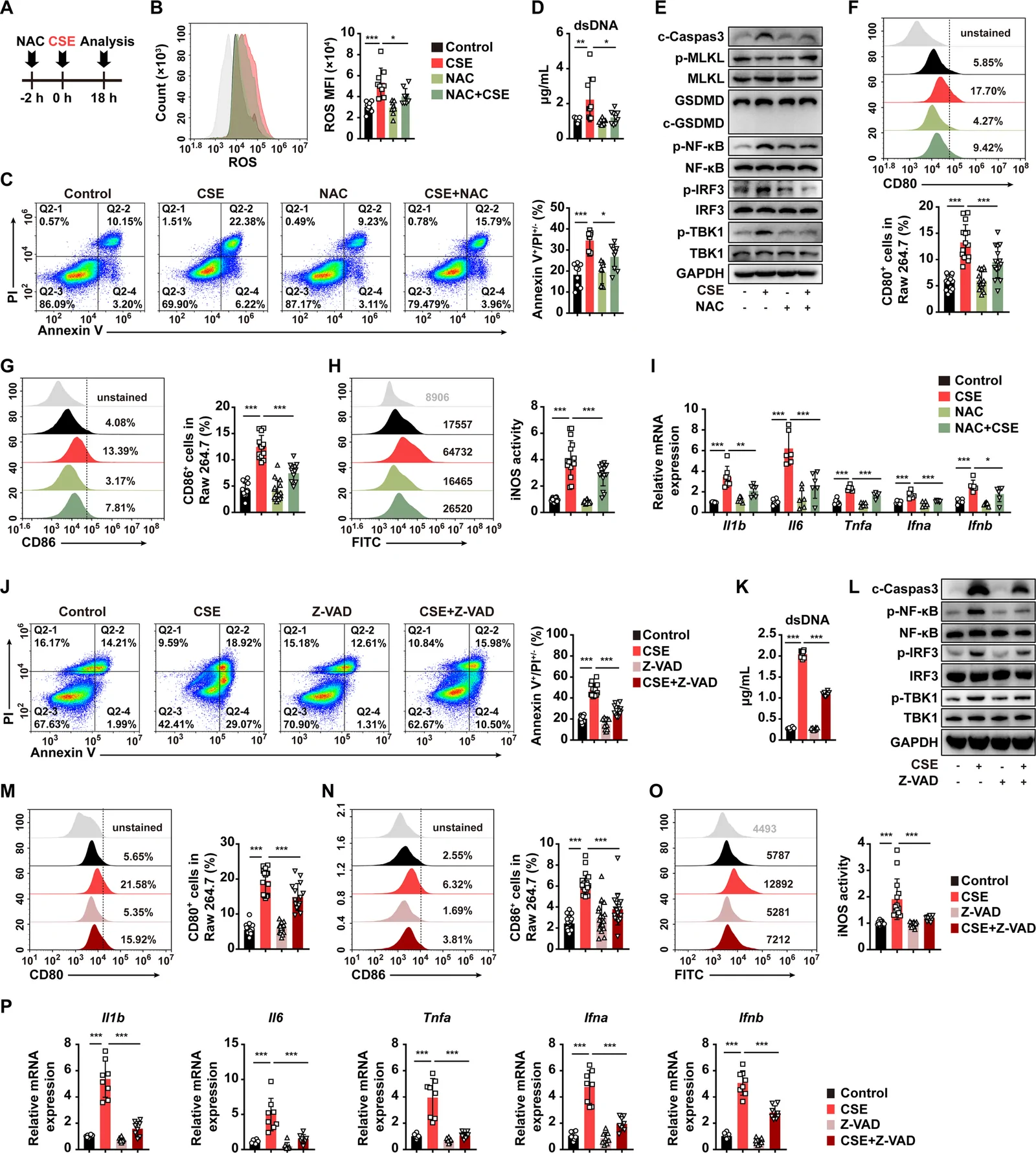
Effects of the antioxidant NAC and apoptosis inhibitor Z-VAD-FMK on dsDNA release during CSE-induced apoptosis in RAW 264.7 cells. (**A**): Schematic of the NAC and CSE treatment strategy in RAW 264.7 cell. (**B**): Flow cytometry analysis of ROS levels after 6 h stimulation (n = 9 per group). (**C**): Flow cytometry analysis of cell death via Annexin V/PI staining (*n* = 9 per group). (**D**): Quantification of dsDNA in the cell culture medium (*n* = 9 per group). (**E**): Western blot analysis of c-Caspase3, p-MLKL, GSDMD, p-NF-κB, p-IRF3 and p-TBK1 protein expression (*n* = 3 per group). Flow cytometry analysis of the percentage of (**F**) CD80+ and (**G**) CD86+ cells, alongside (**H**) iNOS activity (*n* = 14–16 per group). (**I**) RT-qPCR analysis of pro-inflammatory cytokines (Il1b, Il6, Tnfb, Ifna, and Ifnb) mRNA expression in RAW 264.7 cells treated with CSE and NAC (*n* = 6–7 per group). (**J**): Flow cytometry analysis of cell death via Annexin V/PI staining following CSE and Z-VAD-FMK treatment (*n* = 12 per group). (**K**): Quantification of dsDNA in the cell culture medium (*n* = 8 per group). (**L**): Western blot analysis of c-Caspase3, p-TBK1, p-NF-κB and p-IRF3 protein expression (*n* = 3 per group). Flow cytometry analysis of the percentage of (**M**) CD80^+^ and (**N**) CD86^+^ cells, alongside (**O**) iNOS activity; and RT-qPCR analysis of (**P**) inflammatory cytokine (Il1b, Il6, Tnfb, Ifna, and Ifnb) mRNA expression in RAW 264.7 cells treated with CSE and Z-VAD-FMK (*n* = 8–18 per group). Data are presented as mean ± SD; * indicates *p* < 0.05, ** indicates *p* < 0.01, *** indicates *p* < 0.001

Apoptosis, pyroptosis and necroptosis represent distinct pathways of regulated cell death that are linked to DNA damage and strongly associated with cellular stress [40]. Here, we sought to investigate whether CSE-induced oxidative stress drives dsDNA release as a consequence of CSE-triggered cell death. To test this, RAW 264.7 cells were pretreated with either the antioxidant NAC or the pan-caspase inhibitor Z-VAD-FMK for 2 h prior to CSE exposure (Fig. 5A).

Our results demonstrate that ROS levels (Fig. 5B) were elevated by CSE but significantly reduced upon NAC treatment. Correspondingly, CSE exposure significantly increased both the percentage of Annexin V-positive cells (Fig. 5C) and the axtracellular accumulation of dsDNA (Fig. 5D). However, NAC treatment effectively decreased both the percentage of Annexin V-positive cells and dsDNA levels in the CSE plus NAC group compared to the CSE-alone group.

Because extracellular dsDNA can originate from various cell death pathways, we next sought to identify the specific mechanism involved. We evaluated the expression of p-MLKL (a necroptosis marker), cleaved GSDMD (a pyroptosis marker) and cleaved Caspase 3 (an apoptosis marker) in the presence or absence of NAC. Western blot analysis revealed a significant upregulation of c-Caspase 3 upon CS exposure, indicating that apoptosis—rather than necroptosis or pyroptosis—is the primary source of dsDNA. This was supported by the lack of induction or activation of MLKL and GSDMD into their phosphorylated or cleaved forms, respectively.

Furthermore, NAC treatment not only significantly inhibited cleaved Caspase 3 levels but also attenuated the activation of p-NF-κB, p-IRF3 and p-TBK1 in the NAC plus CSE group compared to the CSE-alone group (Fig. 5E). Concurrently, the CSE-induced increases in the percentages of CD80^+^ (Fig. 5F) and CD86^+^ (Fig. 5G) macrophages, downstream pro-inflammatory cytokine expression (*Il1b, Il6, Tnfa, Ifna* and *Ifnb*) (Fig. 5I), and iNOS activity (Fig. 5H) were all markedly suppressed by NAC treatment.

For the in vivo study, mice received NAC once daily prior to the initial CS exposure (Figure S10A). CS stimulation significantly increased ROS generation (Figure S10B), cell apoptosis in the lungs (Figure S10C), and dsDNA production in the BALF (Figure S10D); however, these elevated levels were markedly reduced in the group treated with a combination of NAC and CS. Similarly, NAC prevented the CS-induced increase in both the percentage and absolute number of CD80^+^ (Figure S10E) and CD86^+^ (Figure S10F) macrophages, as well as mean fluorescence intensity of iNOS (Figure S10G) in lung macrophages. Furthermore, NAC suppressed the expression of specific cytokines (IL-1β, IL-6, TNF-α, IFN-α, and IFN-β but not IL-2) (Figure S10H). These findings indicate that NAC suppresses CS-triggered M1 polarization, a process that may be associated with oxidative stress-induced macrophage apoptosis and subsequent dsDNA release.

To further verify whether CSE-induced apoptosis is the primary source of dsDNA release, we utilized the pan-caspase inhibitor Z-VAD-FMK. Accordingly, Z-VAD-FMK dramatically decreased CSE-enhanced cell apoptosis (Fig. 5J) and reduced dsDNA (Fig. 5K) levels in the cell culture medium. Notably, Z-VAD-FMK not only attenuated apoptosis-associated signaling pathways but also inhibited the phosphorylation levels of NF-κB, IRF3 and TBK1 (Fig. 5L). Consistent with our in vivo observations, the changes in the percentage of CD80^+^ (Fig. 5M) and CD86^+^ (Fig. 5N) macrophages, cytokine expression (*Il1b, Il6, Tnfa, Ifna* and *Ifnb*) (Fig. 5P) and iNOS activity (Fig. 5O) in cells exposed to CSE with or without Z-VAD-FMK closely mirrored the results described above.

Taken together, these results demonstrate that CSE triggers oxidative stress to promote macrophage apoptosis, thereby driving enhanced dsDNA release.

### CSE-induced mtDNA release is associated with M1 macrophages polarization in vitro and in vivo

**Fig. 6:**
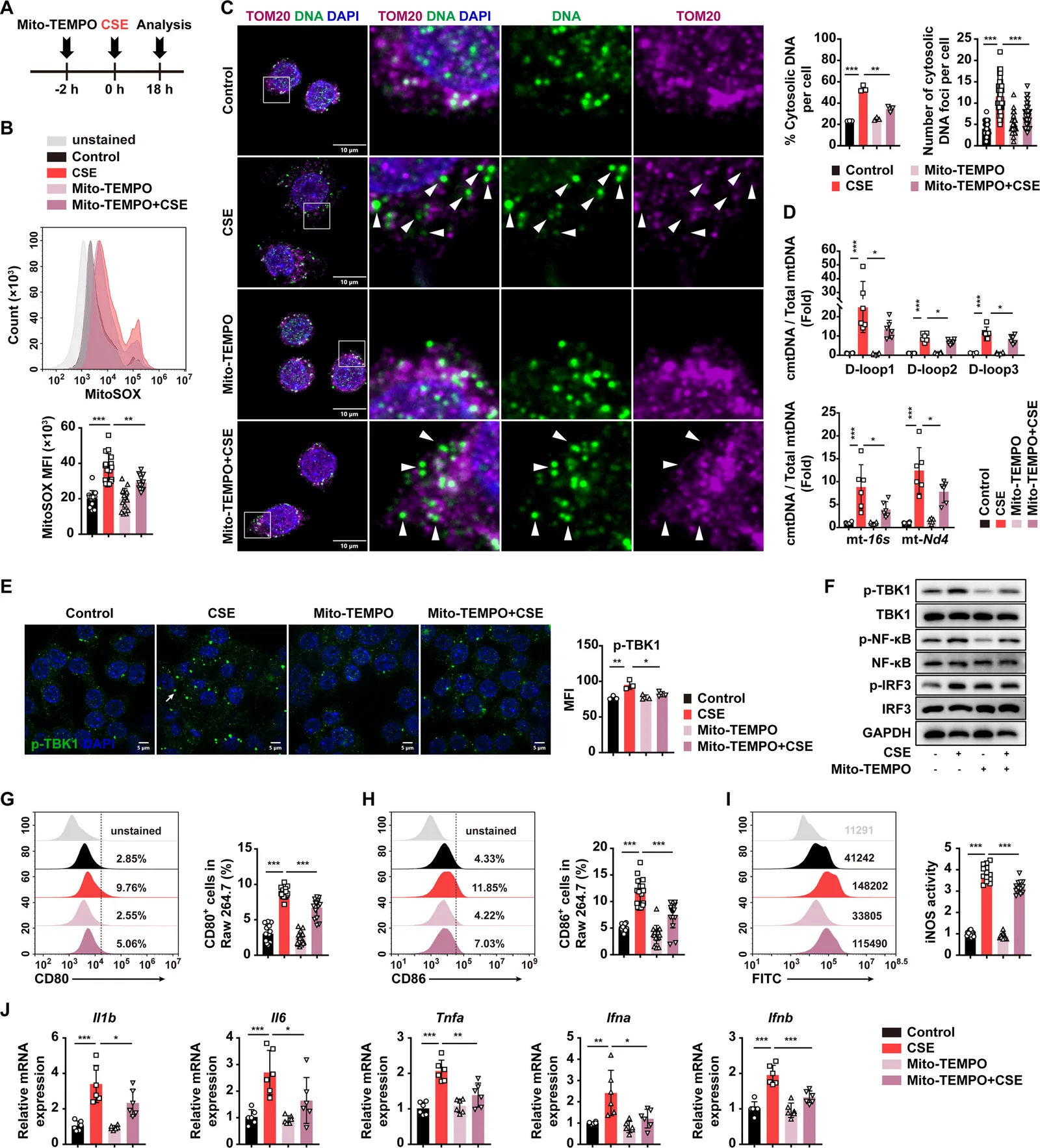
The mitochondrial stress inhibitor Mito-TEMPO mitigates CSE-induced mtDNA release and subsequent polarization of RAW 264.7 cells. (**A**): Schematic of Mito-TEMPO and CSE treatment strategies in RAW 264.7 cells. (**B**): Flow cytometry analysis of mtROS levels following 6 h of stimulation (*n* = 15–16 per group). (**C**): Immunofluorescence detection of mtDNA release into the cytoplasm of RAW 264.7 cells under CSE and Mito-TEMPO treatment (*n* = 3 per group). (**D**): qPCR quantification of cytosolic mtDNA (cmtDNA) markers (D-loop1/2/3, mt-16s and mt-Nd4) under Mito-TEMPO and CSE treatment (*n* = 6 per group). (**E**): Immunofluorescence detection of p-TBK1 expression in RAW 264.7 cells treated with CSE and Mito-TEMPO (*n* = 3 per group). (**F**): Western blot analysis of p-TBK1, p-NF-κB and p-IRF3 signaling protein expressions (*n* = 3 per group). Flow cytometry analysis of the percentage of (**G**) CD80^+^ and (**H**) CD86^+^ cells, alongside (**I**) iNOS activity; and qPCR detection of (**J**) inflammatory cytokine (Il1b, Il6, Tnfb, Ifna, and Ifnb) mRNA expression in RAW 264.7 cells treated with CSE and Mito-TEMPO.(*n* = 6–16 per group) Data are presented as mean ± SD; * indicates *p* < 0.05, ** indicates *p* < 0.01, *** indicates *p* < 0.001

Mitochondrial stress serves not only as a primary source of mtDNA release but also acts as a major driver of COPD pathogenesis [41–43]. Therefore, we sought to determine whether attenuating mitochondrial oxidative stress could mitigate the adverse effects of CSE exposure. To inhibit mitochondrial oxidative stress, RAW 264.7 cells were pretreated with Mito-TEMPO for 2 h prior to CSE exposure (Fig. 6A). As anticipated, this pretreatment significantly decreased the levels of mitochondrial ROS (mtROS) that had been elevated by CSE (Fig. 6B).

Additionally, immune-fluoresce staining suggested that CSE exposure dramatically enhanced the release of mtDNA (green) from the mitochondria into the cytoplasm. Specifically, both the percentage of cytosolic DNA and the number of cytosolic DNA foci per cell were significantly higher in the CSE-treated group compared to the control group (Fig. 6C). Co-treatment with Mito-TEMPO significantly reduced the presence of cytosolic DNA compared to the CSE-alone group (Fig. 6C). These findings were further confirmed by quantifying mtDNA levels in the cytoplasm (Fig. 6D). While CSE exposure significantly elevated the cytosolic levels of D-loop1/2/3, mt-16s and mt-Nd4, Mito-TEMPO treatment alone showed no significant difference compared to the control. Conversely, in the CSE plus Mito-TEMPO group, cytosolic D-loop1/2/3, mt-16s and mt-Nd4 levels were dramatically decreased compared to the CSE group. Collectively, these data suggest that Mito-TEMPO effectively prevents CSE-induced mtDNA release.

We next observed that Mito-TEMPO significantly suppressed the phosphorylation of TBK1 induced by CSE (Fig. 6E-F). Downstream signaling molecules, including p-NF-κB and p-IRF3, were similarly attenuated by Mito-TEMPO (Fig. 6F). Subsequently, we evaluated the expression of M1 macrophage markers and inflammatory mediators. The expression of CD80^+^ (Fig. 6G) and CD86^+^ (Fig. 6H and Figure S11), the production of pro-inflammatory cytokines (*Il1b, Il6, Tnfa, Ifna* and *Ifnb*) (Fig. 6J), and iNOS activity (Fig. 6I) were all dramatically reduced by Mito-TEMPO treatment compared to the CSE-induced levels. Notably, the levels of CD80, CD86, iNOS, and certain cytokines (such as Il1b) remained relatively higher in the Mito-TEMPO plus CSE group than in the control group (Fig. 6G-J). Taken together, these results suggest that mitochondrial stress-triggered mtDNA release is, at least partially, involved in CSE-induced M1 macrophage polarization.

To further verify that mtDNA is associated with CSE-induced M1 macrophages polarization, EtBr was used to deplete mtDNA in RAW 264.7 cells (Figure S12A). As expected, mtDNA levels were significantly decreased (Figure S12B), which led to a relative inactivation of phosphorylated/activated TBK1 (Figure S12C and Figure S7D), as well as the inactivation of NF-κB and IRF3 (Figure S12D). Consequently, we observed that the proportions of CD80^+^ (Figure S12E) and CD86^+^ (Figure S12F) cells, the production of proinflammatory cytokines (*Il1b, Il6, Tnfa, Ifna* and *Ifnb*) (Figure S12H), and iNOS activity (Figure S12G) were significantly suppressed by EtBr treatment despite prior CSE stimulation.

**Fig. 7:**
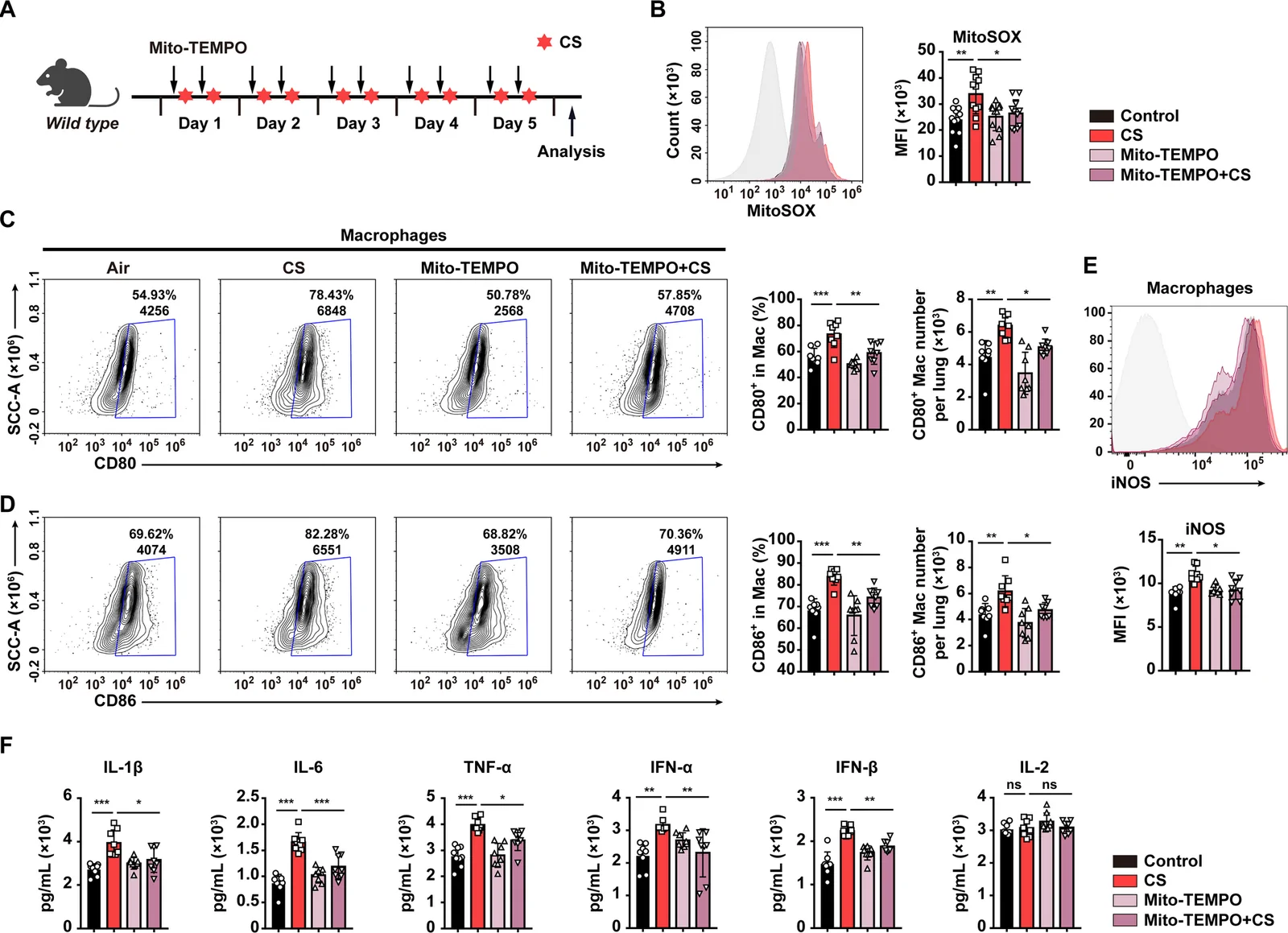
Effects of the mitochondrial stress inhibitor Mito-TEMPO on M1 macrophage polarization in C57BL/6J mice exposed to cigarette smoke (CS). (**A**): Experimental timeline and treatment strategies for Mito-TEMPO and CS exposure in C57BL/6J mice. (**B**): Flow cytometry analysis of mitochondrial stress in lung macrophages using the mitoSOX indicator.(*n* = 11 per group) (**C**) Total cell number and percentage of CD80^+^ and (**D**) CD86^+^ lung macrophages (*n* = 8 per group). (**E**) mean fluorescence intensity of iNOS in lung macrophages. (**F**) Expression levels of inflammatory cytokines (IL-1β, IL-6, TNF-α, IFN-α, IFN-β and IL-2) in lung tissue. Data are presented as mean ± SD; * indicates *p* < 0.05, ** indicates *p* < 0.01, *** indicates *p* < 0.001;

To validate these findings in vivo, mice subjected to CS-exposure were pretreated with Mito-TEMPO, and primary macrophages were subsequently isolated from mice lungs for analysis (Fig. 7A). Consistent with our in vitro data, the CS-induced elevation of mtROS was markedly attenuated by Mito-TEMPO (Fig. 7B). Notably, the in vivo effects of Mito-TEMPO appeared even more pronounced than those observed in vitro, Mito-TEMPO fully neutralized the CS-induced increases in both the percentages and absolute numbers of CD80^+^ (Fig. 7C) and CD86^+^ (Fig. 7D) macrophages, normalized cytokine expression (IL-1β, IL-6, TNF-α, IFN-α, and IFN-β) (Fig. 7F), and significantly decreased the MFI of iNOS positive cells (Fig. 7E) back to baseline control levels.

However, the percentage of CD80^+^ and CD86^+^ macrophages, along with iNOS activity and cytokine levels, remained moderately elevated in the EtBr plus CSE group compared to the control group (Figure S11E-H). This suggests that EtBr didn’t completely abrogate the effects of CSE, potentially because residual dsDNA was not entirely depleted, thereby exerting a persistent, partial stimulatory effect. Overall, these results indicate that mitigating mitochondrial stress with Mito-TEMPO or depleting mtDNA with EtBr attenuates CSE-driven M1 macrophages polarization both in vitro and in vivo via TBK1/NF-κB/IRF3 signaling pathway.

### The dsDNA involved in CSE-induced mitochondrial stress does not depend on cGAS/STING/TBK1 signaling

**Fig. 8:**
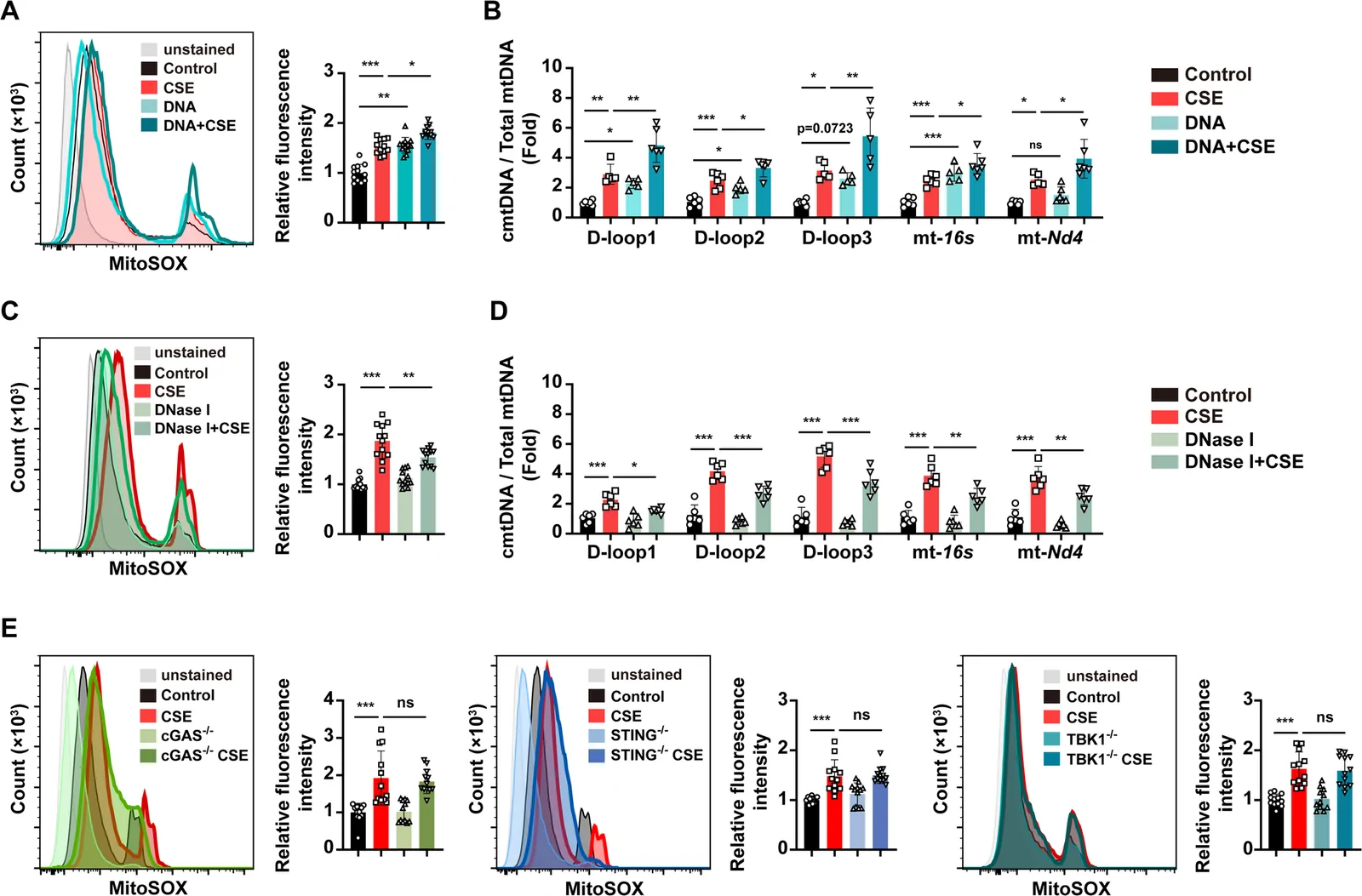
Self-DNA mediates CSE-induced mitochondrial stress independently of the cGAS/STING/TBK1 pathway in RAW 264.7 cells. (**A**): Measurement of mtROS levels via staining and (**B**) quantification of cmtDNA levels via PCR following treatment with CSE, DNA or their combination (*n* = 5–12 per group). (**C**): Measurement of mtROS levels and (**D**) quantification of cmtDNA levels following treatment with CSE, DNase I or their combination (*n* = 6–12 per group). (**E**): Flow cytometry analysis of mtROS levels in CSE-stimulated wild type and cGAS or STING or TBK1-deficient RAW 264.7 cells (*n* = 12 per group). Data are presented as mean ± SD; ns indicates no significant *p* > 0.05, * indicates *p* < 0.05, ** indicates *p* < 0.01, *** indicates *p* < 0.001

We previously confirmed that dsDNA levels are elevated in both patients with COPD and mice exposed to CS. Here, we observed that dsDNA significantly increases mtROS levels to a degree comparable to CSE exposure, with their combination showing a trend toward further enhancement (Fig. 8A). Similarly, dsDNA increased cytosolic mtDNA (cmtDNA) levels, especially D-loop1/2/3 and mt-16s, but not mt-Nd4 (Fig. 8B). to a similar extent as CSE. Notably, the combination of dsDNA and CSE yielded the highest cmtDNA levels across all experimental groups.

To investigate further, we treated cells with DNase I. While DNase I alone did not alter baseline mtROS (Fig. 8C) or cmtDNA (Fig. 8D) levels, it effectively attenuated the CSE-induced elevations in both parameters .Furthermore, the deficiency of cGAS, STING or TBK1 in RAW 264.7 cells did not affect CSE-induced mtROS production (Fig. 8E). Taken together, these results indicate that while dsDNA mediates CSE- induced mitochondrial stress, this process occurs independently of the cGAS/STING/TBK1 pathway.

In summary, this study demonstrates that cigarette smoke induces mitochondrial stress, leading to the overproduction of ROS and the subsequent release of dsDNA. Both extracellular dsDNA and leaked mtDNA can activate downstream cGAS signaling, which in turn triggers the STING/TBK1 axis. This cascade activates the transcription factors IRF3 and NF-κB, ultimately upregulating the expression of CD80, CD86, iNOS, and pro-inflammatory cytokines (*Il1b, Il6, Tnfa, Ifna* and *Ifnb*), thereby driving M1 macrophages polarization (Fig. 8F).

## Discussion

Cigarette smoke (CS), an environmental hazard, is heavily implicated in the pathogenesis of COPD. Globally, COPD stands alongside cancer and cardiovascular diseases as one of the leading inflammatory lung conditions [43–46]. Neutrophil extracellular traps (NETs) have been closely linked to CS-triggered lung inflammation and subsequent COPD progression [47, 48]. Notably, patients with COPD exhibit heightened CSE-associated neutrophils NETosis, alongside an increased involvement of mtDNA within these NETs [47]. Furthermore, CSE-NETs-induced DCs maturation depends on the mtDNA sensor cGAS via NF-κB signaling [47]. Recent literature highlights the cGAS-STING pathway as a cornerstone of innate immunity, mapping its pathological over activation across a spectrum of chronic inflammatory lung diseases, including pulmonary fibrosis, asthma, and chronic obstructive pulmonary disease (COPD) [49]. While the established literature broadly links excessive STING activation to tissue damage, airway hyper reactivity, and diminished respiratory function, the precise upstream triggers and downstream cellular orchestrators driving these phenotypes have remained largely elusive [49]. In the present study, we verified that cell-free dsDNA is significantly elevated in both COPD patients and preclinical mouse models, aligning with the real-world data outlined above. Our findings also indicate that activation of the cGAS/STING/TBK1 signaling ultimately drives pro-inflammatory M1 macrophage reprogramming and type I interferon immune response.

CSE can aberrantly accelerate cell death, prompting the release of extracellular dsDNA driven by CSE-induced ROS. Concurrently, it stimulates intracellular mtDNA overproduction mediated by mtROS. Our results demonstrate that both intracellular and extracellular dsDNA levels increase significantly under CS exposure. This freely circulating dsDNA rapidly transmits signals throughout the immune system, amplifying a systemic chain reaction. Because cGAS signaling is a critical mediator of the inflammatory innate immune response [50]. Inhibiting the cGAS/STING pathway attenuates inflammation [51] and may offer novel therapeutic avenues for COPD [52]. Consequently, we propose that targeting cGAS-related signaling networks holds a strong potential as a therapeutic strategy for CS-induced lung disease.

Oxidative stress, characterized by an accumulation of excess ROS, serves as a primary mechanism driving COPD pathogenesis [43, 53]. Early-stage diagnosis of COPD remains a formidable challenge, despite the clinical utility of systemic biomarkers such as C reactive protein (CRP), IL-6, IL-8 etc [54]. Several inflammation-associated pathways—including NF-κB, cGAS/STING, STAT, and Smads—have been well-documented for their roles in lung pathology [43]. Clinically, a cohort study (NCT01969344) indicated that urine mtDNA levels could serve as a diagnostic marker for COPD severity, displaying sex-specific differences where females presented with higher urine mtDNA levels and more severe respiratory symptoms than males [55]. Additionally, a clinical cohort of 949 patients with moderate-to-severe COPD revealed that elevated circulating dsDNA levels are associated with a higher risk of mortality [32]. Taken together with our experimental observations, these data suggest that monitoring circulating dsDNA levels provides a critical parameter for COPD diagnosis.

Cigarette smoke (CS) induces mitochondrial damage tied to mtROS production and subsequent COPD pathogenesis [56]. Elevated mtROS levels and the concomitant release of mtDNA are known to engage cGAS/STING signaling to mediate inflammatory immune responses [57], while excessive mtROS can upregulate the mtDNA copy number under oxidative stress [58]. Although airway macrophages are vital for maintaining lung homeostasis and responding to inhaled particulate matter [59], the precise mechanism by which the interaction among CSE, dsDNA and intrinsic macrophage cGAS/STING signaling contributes to lung inflammation has remained elusive. As the primary organelles responsible for managing oxidative stress and immune responses, mitochondria overproduce ROS under stress, leading to the leakage of dsDNA and mtDNA. A failure to clear these impaired mitochondria can trigger cascading inflammatory cascades [60]. Our results from a CS-induced COPD model elucidate that elevated dsDNA levels drive both type-I IFN production and NF-κB-mediated pro-inflammatory cytokine activity, promoting M1 reprogramming via the cGAS/STING/TBK1 pathway. Moreover, mtDNA within CSE-induced NETs promotes IL-1β, NF-κB-dependent cytokines, and autoimmune responses through cGAS/TLR9 signaling in CS-induced COPD [47]. This collective evidence confirms that the cGAS signaling network acts as a central hub for the inflammatory immune response. Herein, we demonstrate that downregulating cGAS, STING and TBK1 significantly attenuates p-TBK1 activation and dampensthe macrophage M1 polarization otherwise exacerbated by CSE exposure.

A wide range of human diseases are associated with dsDNA, where the accumulation or overproduction of dsDNA serves as a key indicator of pathological stress. Previous studies indicate that Tom20-positive mitochondrial membrane can facilitate the leakage of mtDNA into the cytosol [51]. This aligns with our finding that CSE stimulation significantly enhances both mtDNA release and the subsequent macrophage immune response. Mechanistically, we verified that mitochondrial ROS scavenger Mito-TEMPO dramatically attenuated CSE-induced extracellular mtDNA release. Furthermore, both Mito-TEMPO and EB significantly reversed CSE-triggered macrophage M1 reprogramming and pro-inflammatory cytokine expression. Maintaining mitochondrial homeostasis is critical for suppressing ROS generation; this oxidative stress, elevated by CSE, is strongly linked to the pathogenesis of CSE-induced COPD [61]. Consequently, reducing dsDNA levels via DNase I treatment, mitigating ROS or apoptosis levels, and modulating mitochondrial function can effectively suppress the M1 pro-inflammatory response. Indeed, both in vivo and in vitro experiments confirmed that DNase I treatment significantly decreased CS/CSE-triggered M1 polarization and pro-inflammatory cytokines expression, a process mediated by the TBK1-IRF3-NF-κB signaling axis (Fig. 4). Moreover, our results suggest that targeting and mitigating CSE-enhanced ROS could serve as a viable therapeutic strategy for CSE-associated diseases (Fig. 5). Data obtained using the antioxidant NAC and apoptosis inhibitor Z-VAD-FMK indicates that CSE induced apoptosis—rather than pyroptosis or necroptosis–is the primary contributor to dsDNA release (Fig. 5). Additionally, while VDAC1 may play a vital role in dsDNA transport and ROS generation [9, 42] during this process, we did not explore this mechanism further; this remains a promising avenue for a more in-depth investigation in future studies.

We observed that CSE-induced mtROS production is promoted by DNA co-stimulation. While the addition of DNase I partially attenuates the effects of CSE, mtROS levels remain elevated. This suggests that DNase I might not completely degrade cytosolic dsDNA, or alternatively other nucleic acid species capable of triggering an mtROS response are present but resistant to DNase I cleavage. Notably, several studies have implicated the cGAS/STING/TBK1 pathway in ROS and mtROS generation [62–64]. For instance, a study on hepatocellular carcinoma cells showed that increased mtROS is associated with the absence of cGAS, with the authors emphasizing the critical role of outer-mitochondria-membrane-localized cGAS [63]. Based on this, we hypothesized that cGAS might recognize cytosolic dsDNA and traffic it to the mitochondria, thereby driving excessive ROS production. However, our data demonstrated that CSE-induced mtROS generation occurs independently of the cGAS/STING/TBK1 pathway (Fig. 8), indicating that this specific axis is not required for CSE-triggered mtROS production. In contrast, DNA significantly enhances CSE-induced type I IFN response and M1 macrophage polarization via a TBK1-dependent mechanism involving the activation of NF-κB and IRF3 (Figure S4).

In summary, this study demonstrates that the CSE-triggered immune response is driven by an overproduction of dsDNA resulting from excessive ROS. This accumulation activates cGAS/STING/TBK1 signaling, ultimately leading to M1 macrophage polarization and a type I interferon immune response. Consequently, targeting the cGAS/STING/TBK1 signaling or reducing ds/mtDNA overproduction can attenuate M1 macrophage polarization. Therapeutic strategies simultaneously targeting the cGAS/STING/TBK1 axis and mitochondria dysfunction represent a promising next-generation approach against inflammatory lung diseases and warrant further exploration.

## Conclusions, Limitations, and perspectives

While the existing literature establishes the cGAS-STING pathway as a macro-level driver of chronic lung diseases [49], this study elucidates the underlying micro-level mechanics. By mapping the specific CSE-mtROS-mtDNA-cGAS/STING/TBK1-M1 macrophage and CS-ROS-apoptosis-dsDNA-cGAS/STING/TBK1-M1 macrophage axis, these findings transition the field from broad phenotypic observation to precise, targeted therapeutic design, offering a distinct structural blueprint for mitigating environmental-hazard-induced pulmonary decline.

Characterization across all treatment strategies revealed that in vitro RAW 264.7 cells exhibited varying sensitivities regarding their M1 phenotypic patterns. Overall, NAC demonstrated the highest efficacy in modulating M1 macrophage polarization, whereas Z-VAD-FMK exerted relatively weaker effects. The mitochondrial stress inhibitor Mito-TEMPO and DNase I were the least effective across all evaluated markers. These findings indicate that broader-target interventions, such as NAC and Z-VAD-FMK, exert more robust effects than highly specific agents like DNase I and Mito-TEMPO. NAC and Z-VAD-FMK are highly efficient might due to they intercept primary, upstream intracellular drivers of the cigarette smoke extract (CSE) response, effectively halting the initial smoke-induced oxidative burst and the cell death procedure. Conversely, Mito-TEMPO and DNase I target downstream or compartmentalized elements—namely, mitochondrial-specific matrix radicals and extracellular dsDNA—that occur too late or are too localized to prevent global transcription networks from steering the macrophage toward an M1 phenotype. Regarding downstream signaling, cGAS knockdown overall exerted a stronger suppressive effect on M1 macrophage polarization than downregulated STING or TBK1 in vitro (Figure S14).

This study has a few notable limitations that should be acknowledged:

1. Sample size and study duration: The patient sample size is relatively small, and the current in vivo investigation focuses exclusively on short-term outcomes. Consequently, changes in the lung tissue inflammatory index were difficult to detect.
2. Macrophage polarization scope: The analysis primarily focuses on M1 macrophage polarization, offering only a partial assessment of M2 polarization. Furthermore, *Lyz2*^*cre*^ mediated deletion of *Cgas* or *Tbk1* is not strictly macrophage-intrinsic; it can also affect neutrophils, monocytes, and dendritic cells due to off-target deletion and we didn’t analyze all of them. However, our findings suggest that cigarette smoke (CS) does not alter the frequencies of neutrophils, or activated CD86^+^ dendritic cells.
3. Cellular subtype resolution: Due to experimental constraints, we were unable to conduct an in-depth, isolated analysis of AM and IM from the mice lung samples. Instead, our findings reflect overall, total macrophage population.

To address these distinct mechanistic gaps, more comprehensive, long-term and in-depth investigations are required. Targeted studies addressing these specific resolutions are currently underway within our future research framework.

## Supplementary Information

Supplementary Material 1.

Supplementary Material 2.

## Data Availability

No datasets were generated or analysed during the current study.
