# Supplementary material for: Self-DNA mediates tobacco-induced lung inflammation via macrophage-intrinsic cGAS/STING/TBK1 signaling
Source: Cell Commun Signal. 2026 Aug 10;24:607. doi: 10.1186/s12964-026-03124-3 (PMC13761130; doi:10.1186/s12964-026-03124-3)
Supplement: Supplementary file 1 [file 12964_2026_3124_MOESM1_ESM.pdf]

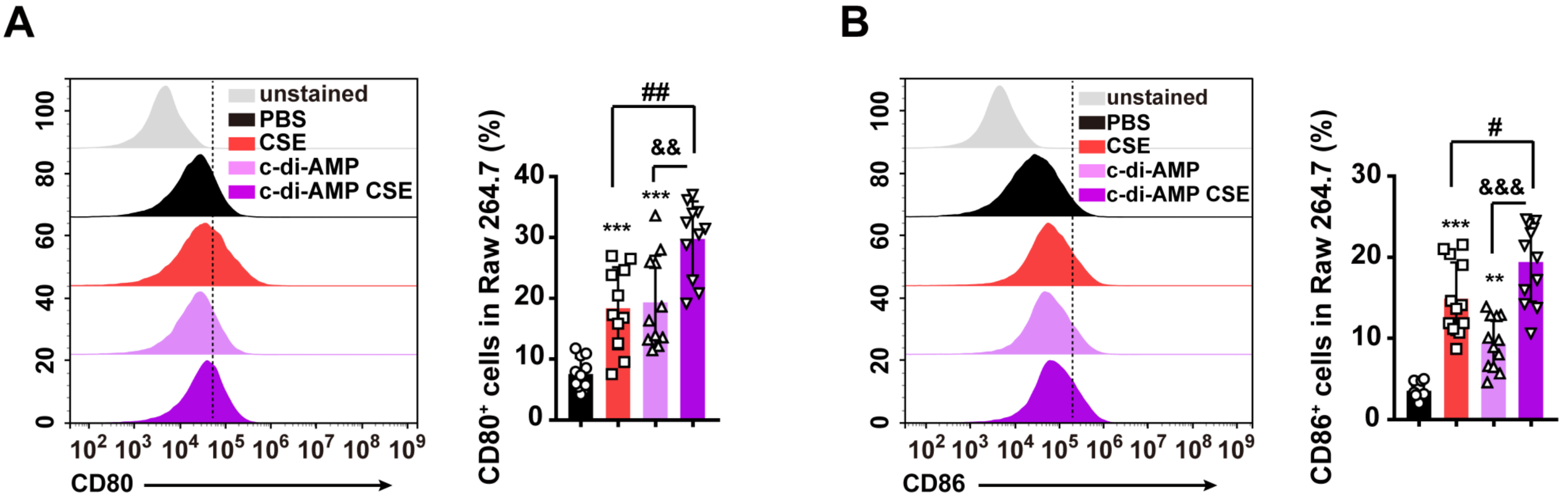

Fig. S1 Effects of c-di-AMP on CD86 and CD80 expression in CSE-induced RAW 264.7 cells
(**A-B**): Flow cytometry analysis of the percentage of (**A**): CD86$^+$ cells and (**B**): CD80$^+$ cells following treatment with CSE in the presence or absence of c-di-AMP (n = 11-12 per group). Data are presented as mean ± SD; ** indicates $p < 0.01$, *** indicates $p < 0.001$ compared with PBS group; $^{\#}$ indicates $p < 0.05$, $^{\#\#}$ indicates $p < 0.01$ compared with CSE group; $^{\&\&}$ indicates $p < 0.01$, $^{\&\&\&}$ indicates $p < 0.001$ compared with c-di-AMP group.

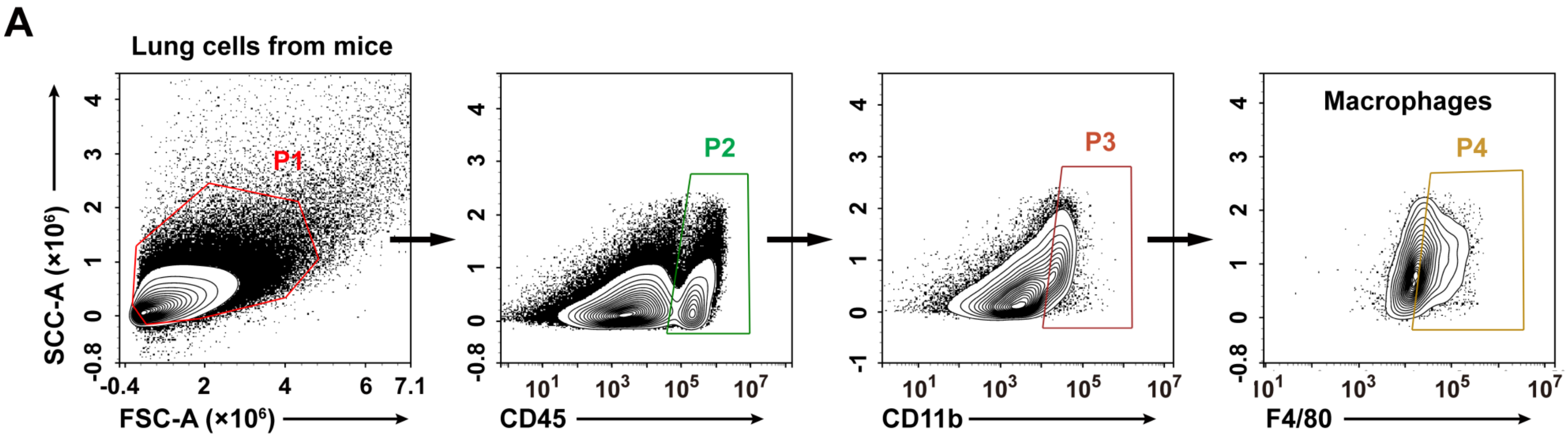

Fig. S2 Flow cytometry gating strategy for macrophage identification

(**A**): Flow cytometry gating strategy for mice lung macrophage: $CD45^{+}F4/80^{+}CD11b^{+}$.

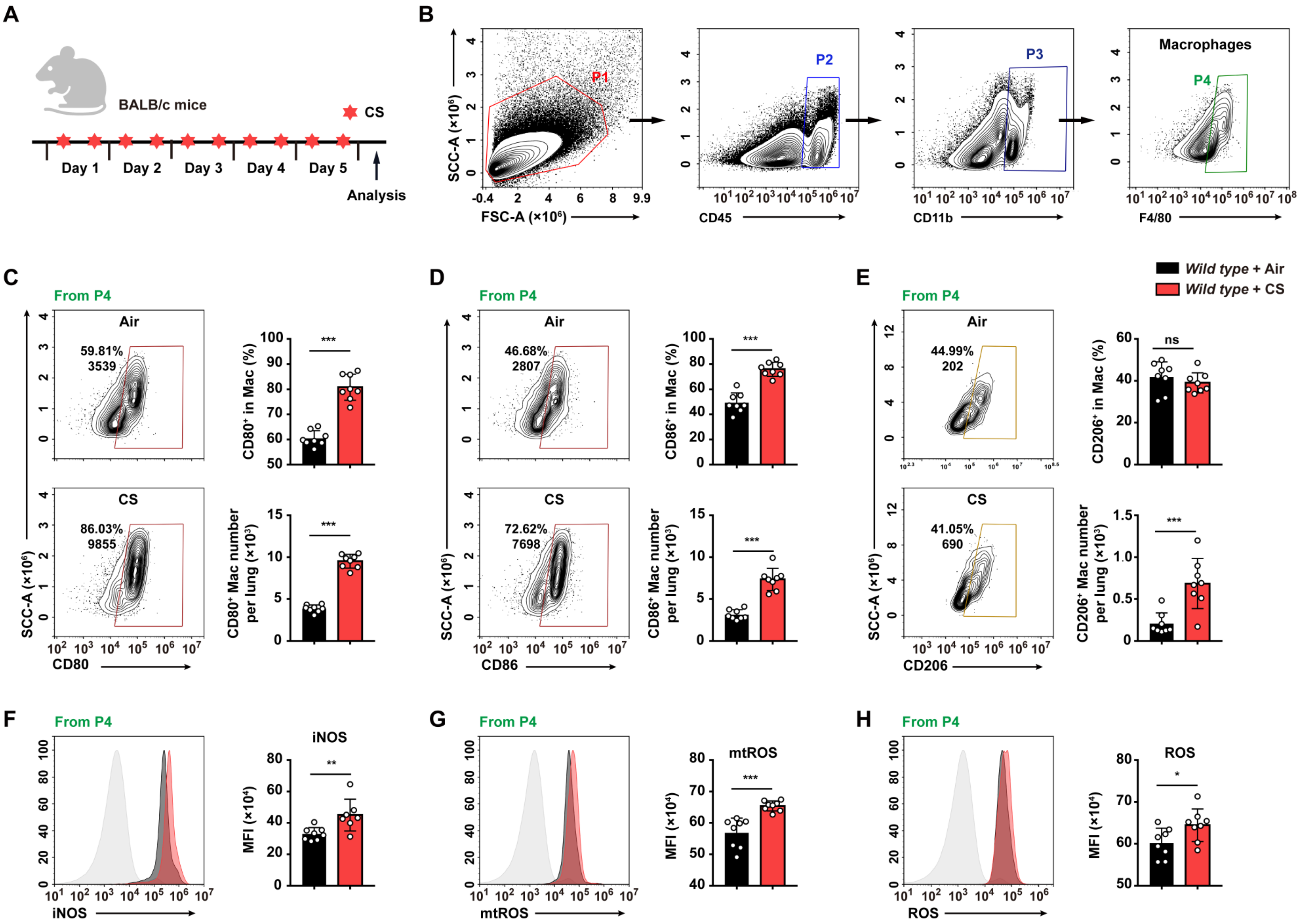

Fig. S3 Cigarette smoke (CS) exposure induces M1 macrophages polarization, oxidative stress and mitochondrial stress in BALB/c mice

(**A**): Experimental schema: BALB/c mice were exposed to CS twice daily for five consecutive days, with lung tissue harvested for analysis on day 6 (n = 8 per group). (**B**): Flow cytometry gating strategy: Identification of lung macrophages using the $CD45^+CD11b^+F4/80^+$ phenotype. (**C-E**): Absolute cell counts and percentages of (**C**) $CD80^+$, (**D**) $CD86^+$, and (**E**) $CD206^+$ macrophage subsets in murine lung tissue. (**F-H**): Flow cytometry analysis mean fluorescence intensity of (**F**) iNOS, (**G**) mtROS, and (**H**) total ROS levels within lung macrophage populations. Data are presented as mean ± SD; * indicates $p < 0.05$, ** indicates $p < 0.01$, *** indicates $p < 0.001$, ns indicates no statistical difference.

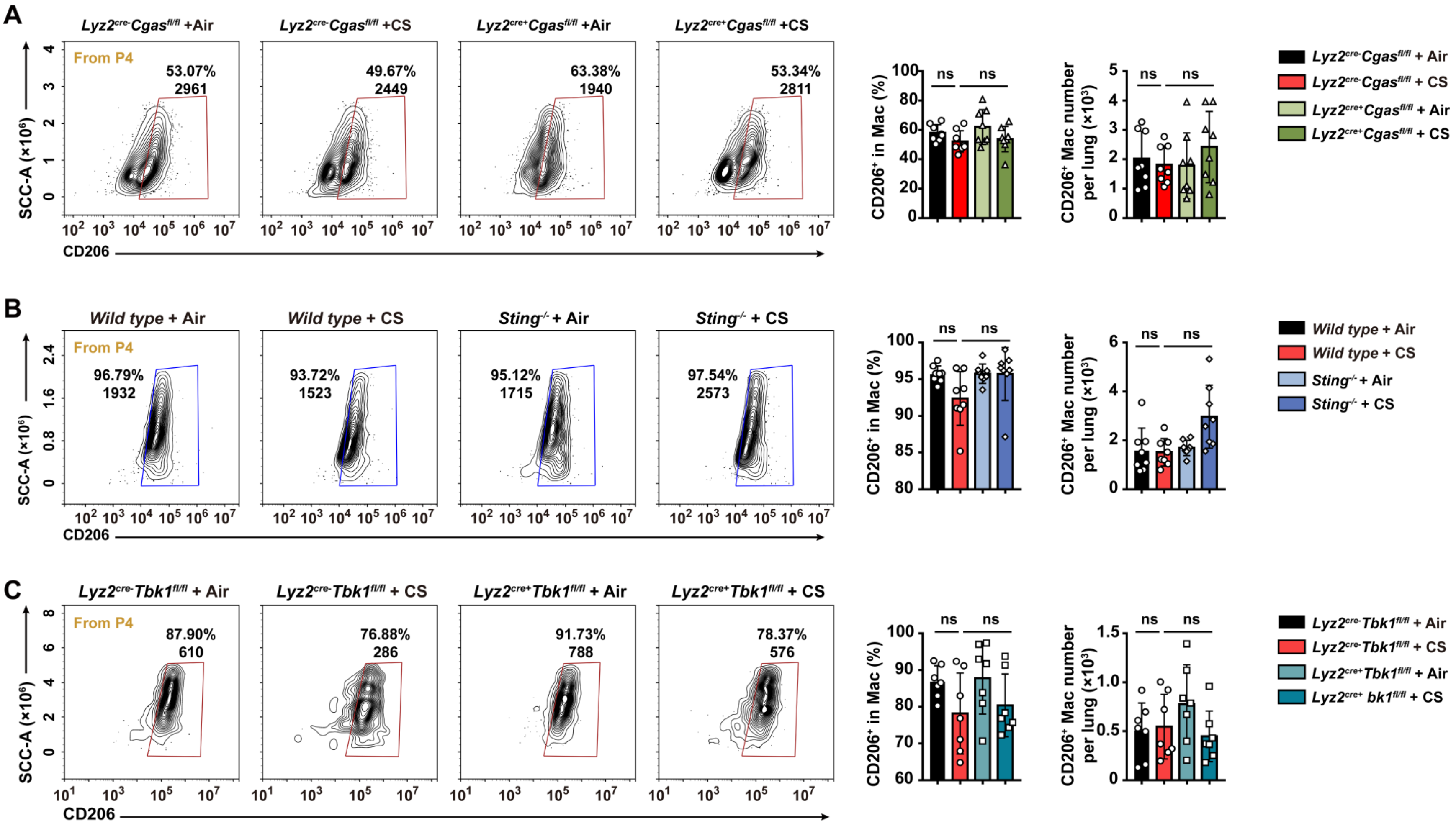

Fig. S4 Cigarette smoke (CS) effects on lung macrophage M2 polarization via cGAS/STING/TBK1 pathway

Continued from Figure 3, wild type, *Lyz2*$^{cre-}$*Cgas*$^{fl/fl}$, *Lyz2*$^{cre-}$*Tbk1*$^{fl/fl}$, *Lyz2*$^{cre+}$*Cgas*$^{fl/fl}$, *Lyz2*$^{cre+}$*Tbk1*$^{fl/fl}$ and *Sting*$^{-/-}$ mice were exposed to CS twice daily for five consecutive days. Lung tissue samples were harvested on day 6 for downstream analysis. Gating strategy for lung macrophage flow cytometry: CD45^{+}F4/80^{+}CD11b^{+}. (**A-C**): Absolute cell counts and percentage of CD206^{+} M2 macrophages are shown for the lung tissues of (**A**) *Lyz2*$^{cre-}$*Cgas*$^{fl/fl}$ and *Lyz2*$^{cre+}$*Cgas*$^{fl/fl}$, (**B**) wild type and *Sting*$^{-/-}$ and (**C**) *Lyz2*$^{cre-}$*Tbk1*$^{fl/fl}$ and *Lyz2*$^{cre+}$*Tbk1*$^{fl/fl}$ mice. Data are presented as mean ± SD; ns indicates no statistical difference.

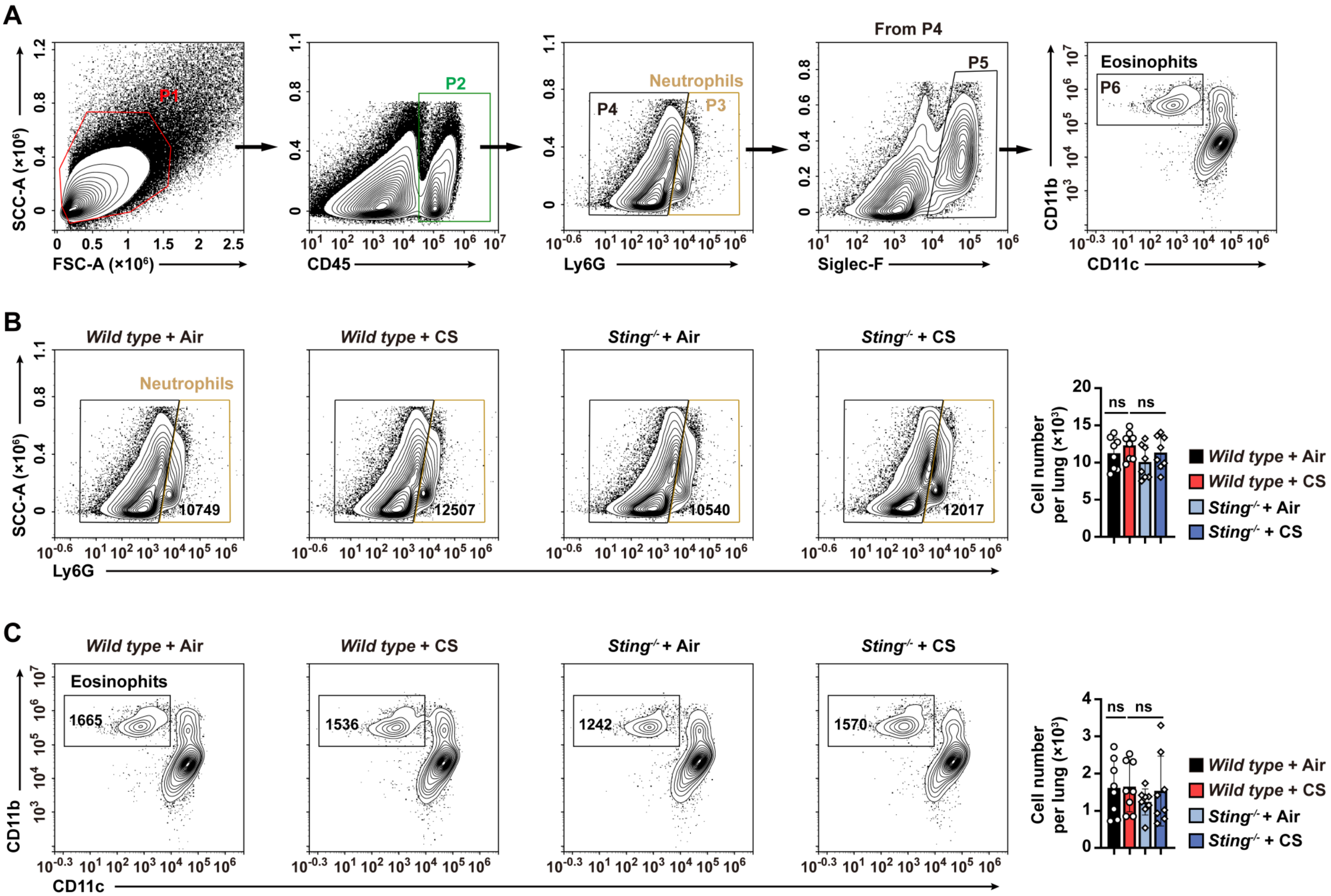

Fig. S5 Flow cytometry analysis of lung neutrophils and eosinophils in *Sting*$^{-/-}$ mice

(**A**): Representative flow cytometry gating strategy for isolating lung neutrophils and eosinophils (n = 8 per group). (**B-C**): Absolute counts of (**B**) neutrophils and (**C**) eosinophils in lung tissues isolated from wild type and *Sting*$^{-/-}$ mice. Data are presented as mean ± SD; ns indicates no statistical difference.

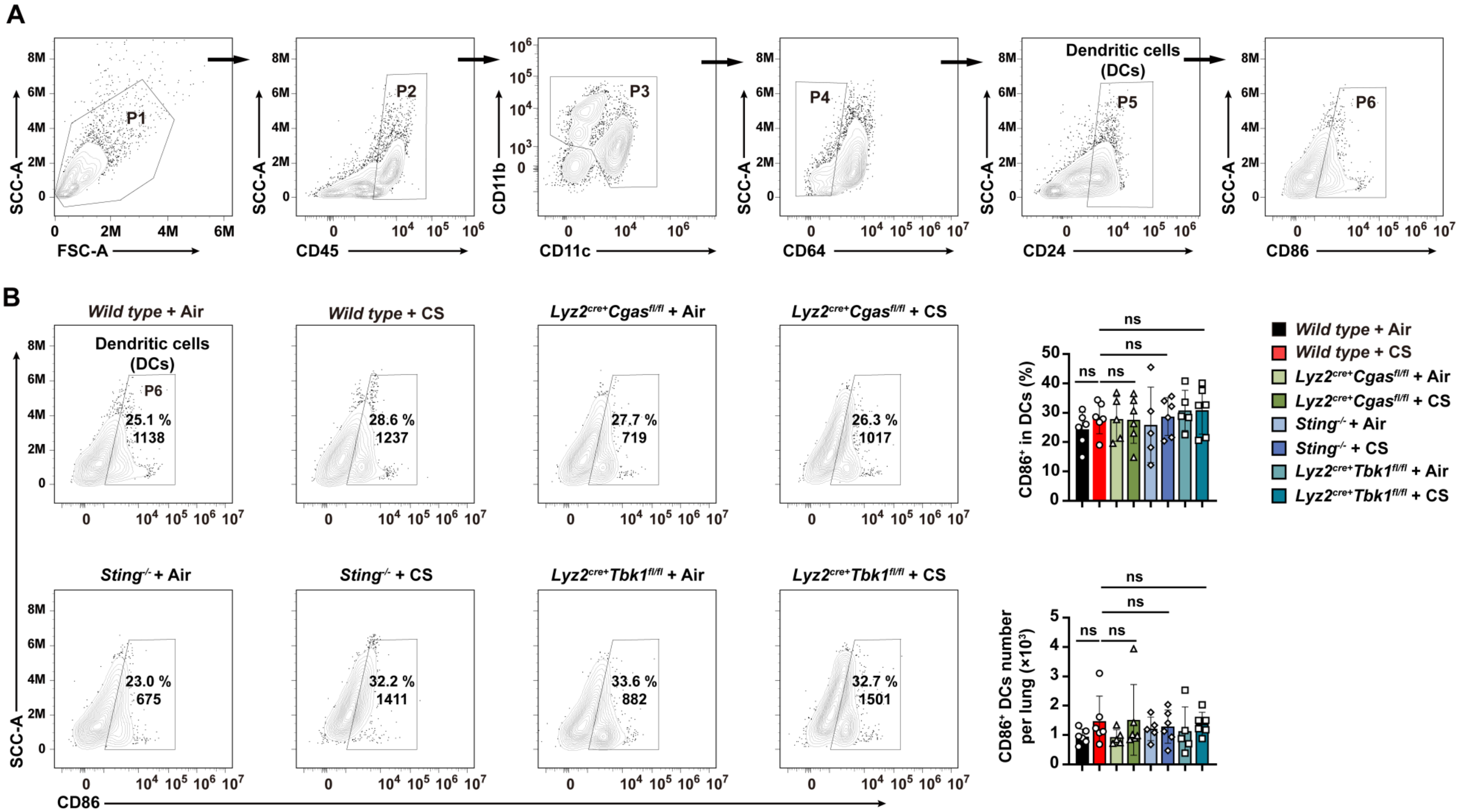

Fig. S6 Flow cytometry analysis of lung CD86$^+$ DCs in genetic modified mice

(**A**): Representative flow cytometry gating strategy for isolating lung dendritic cells (DCs) (n = 5-6 per group). (**B**): Absolute counts and percentages of DCs in lung tissues isolated from wild type and gene modified mice. Data are presented as mean ± SD; ns indicates no statistical difference.

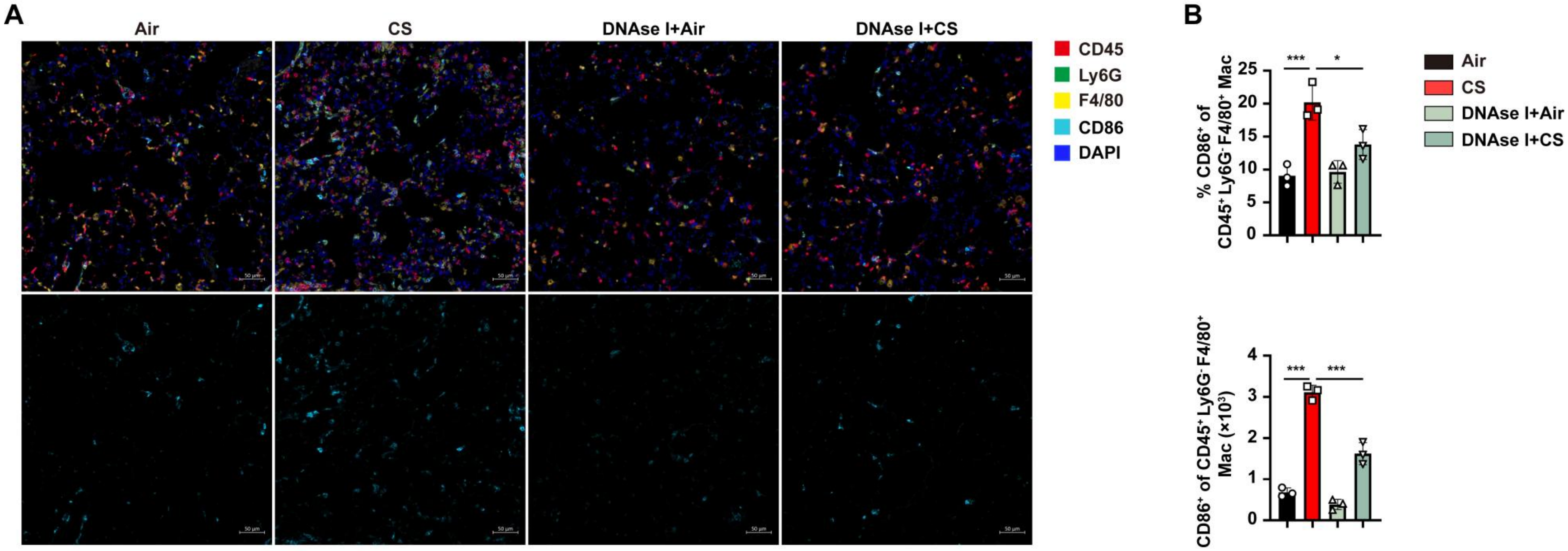

Fig. S7 DNase I attenuates CSE-induced inflammatory M1 macrophage
(**A-B**): Multiplexed immunohistochemistry of $CD86^+$ macrophage showing percentage (**A**) and absolute cell counts (**B**) in the presence and absence of DNase I in mice (n = 3 per group). Data are presented as mean ± SD; * indicates $p < 0.05$, *** indicates $p < 0.001$.

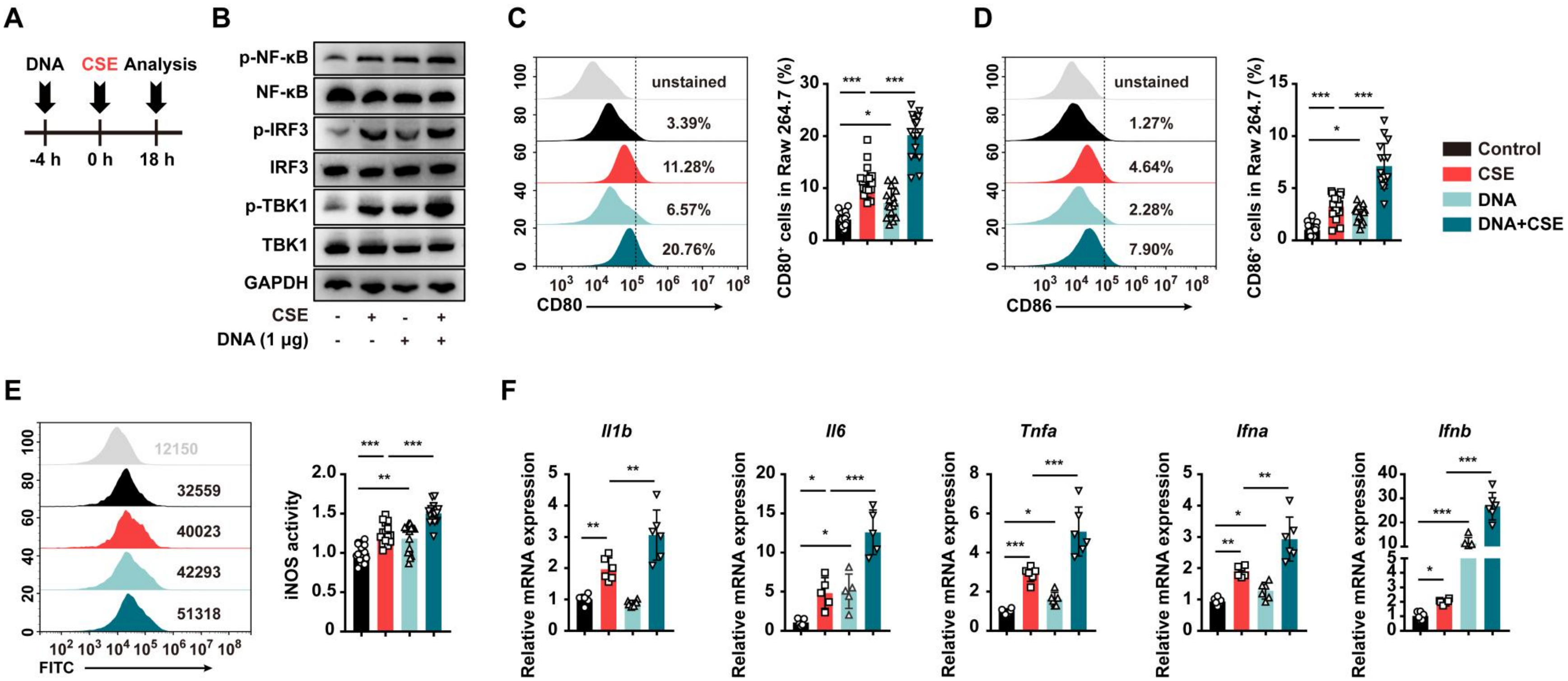

Fig. S8 Effects of extracellular DNA on M1 macrophage polarization and the cGAS/STING/TBK1 pathway in CSE-exposed RAW 264.7 cells

(**A**): Schematic representation of the treatment strategies using CSE and extracellular DNA (total extracellular DNA was isolated and purified from CSE treated RAW264.7 cells using a Vazyme kit). (**B**): Western blot analysis of p-TBK1, p-NF-κB and p-IRF3 activation levels (n = 3 per group). (**C-E**): Flow cytometry analysis of RAW 264.7 cells following CSE and extracellular DNA exposure, showing the percentages of (**C**) $CD80^+$ and (**D**) $CD86^+$ cells, along with (**E**) iNOS activity (n = 15-16 per group). (**F**): RT-qPCR analysis of inflammatory cytokine (*Il1b*, *Il6*, *Tnfa*, *Ifna*, and *Ifnb*) mRNA expression levels in RAW 264.7 cells subjected to CSE and extracellular DNA (n = 5-6 per group). Data are presented as mean ± SD; * indicates $p < 0.05$, ** indicates $p < 0.01$, *** indicates $p < 0.001$.

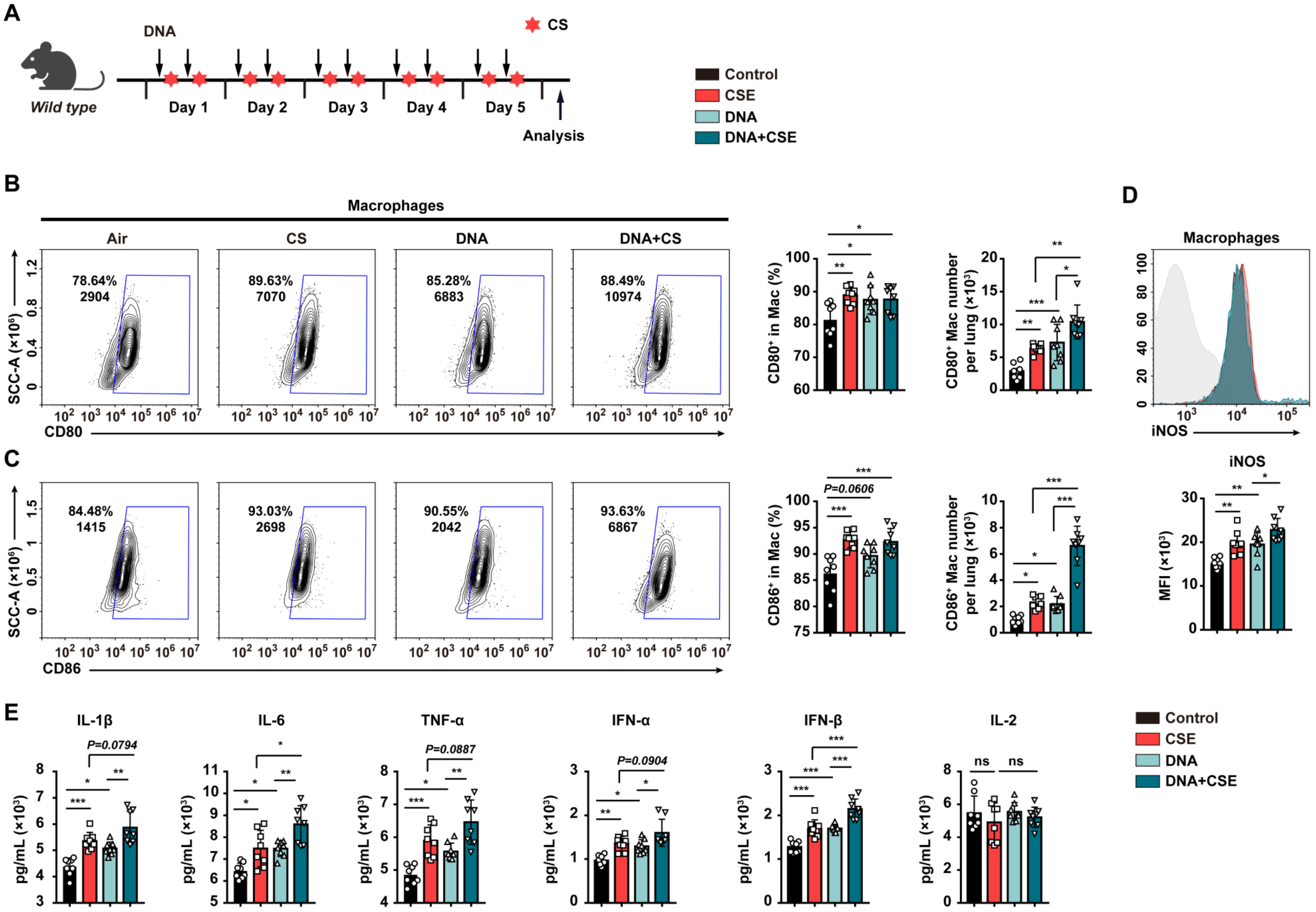

Fig. S9 Effects of exogenous DNA on inflammatory response and lung macrophages polarization in C57BL/6J mice exposed to CS

(**A**): Experimental timeline and strategies for exogenous DNA treatment and CS exposure in C57BL/6J mice (n = 8 per group). (**B-C**): Flow cytometry analysis of (**B**) $CD80^+$ and (**C**) $CD86^+$ macrophage cell counts and percentages in mouse lung tissue. (**D**): Mean fluorescence intensity of iNOS in lung macrophages. (**E**): ELISA quantification of inflammatory cytokines (IL-1β, IL-6, TNF-α, IFN-α, IFN-β and IL-2) concentrations in mouse lung tissue. Data are presented as mean ± SD; * indicates $p < 0.05$, ** indicates $p < 0.01$, *** indicates $p < 0.001$.

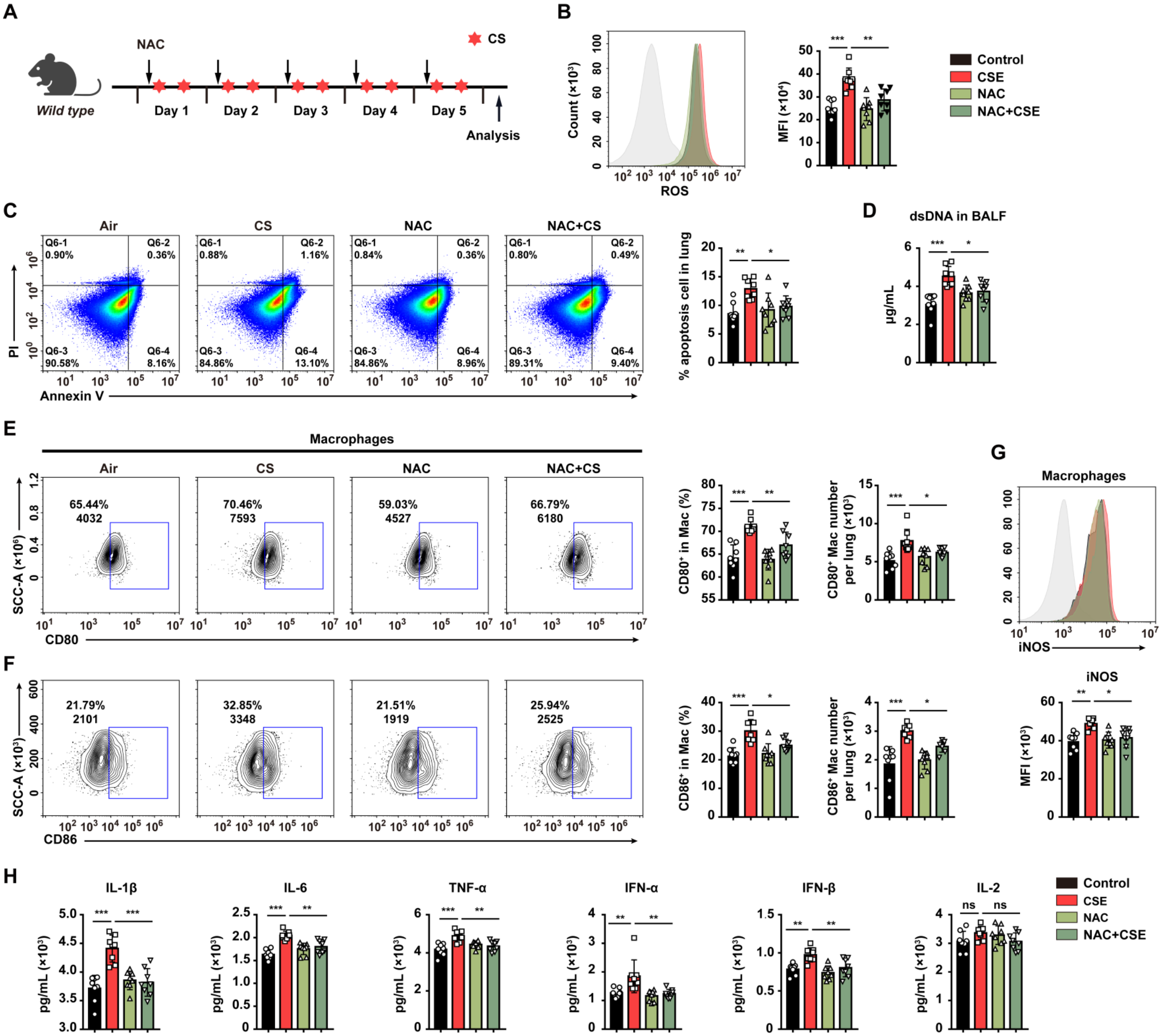

Fig. S10 Effects of the antioxidant drug NAC on mice exposed to CS

(**A**): Experimental schema of NAC and CS treatment regiments in C57BL/6J mice (n = 7-8 per group). (**B**): Flow cytometry evaluation of ROS production. (**C**): Flow cytometry analysis of cellular apoptosis. (**D**): Quantification of dsDNA in bronchoalveolar lavage fluid (BALF). (**E-F**): Flow cytometry analysis of the total count and proportion of (**E**) CD80^{+} and (**F**) CD86^{+} macrophages in murine lung tissue. (**G**): Mean fluorescence intensity of iNOS in lung tissue macrophages. (**H**): ELISA quantification of inflammatory cytokines (IL-1β, IL-6, TNF-α, IFN-α, IFN-β and IL-2) concentrations in murine lung tissue. Data are presented as mean ± SD; * indicates $p < 0.05$, ** indicates $p < 0.01$, *** indicates $p < 0.001$.

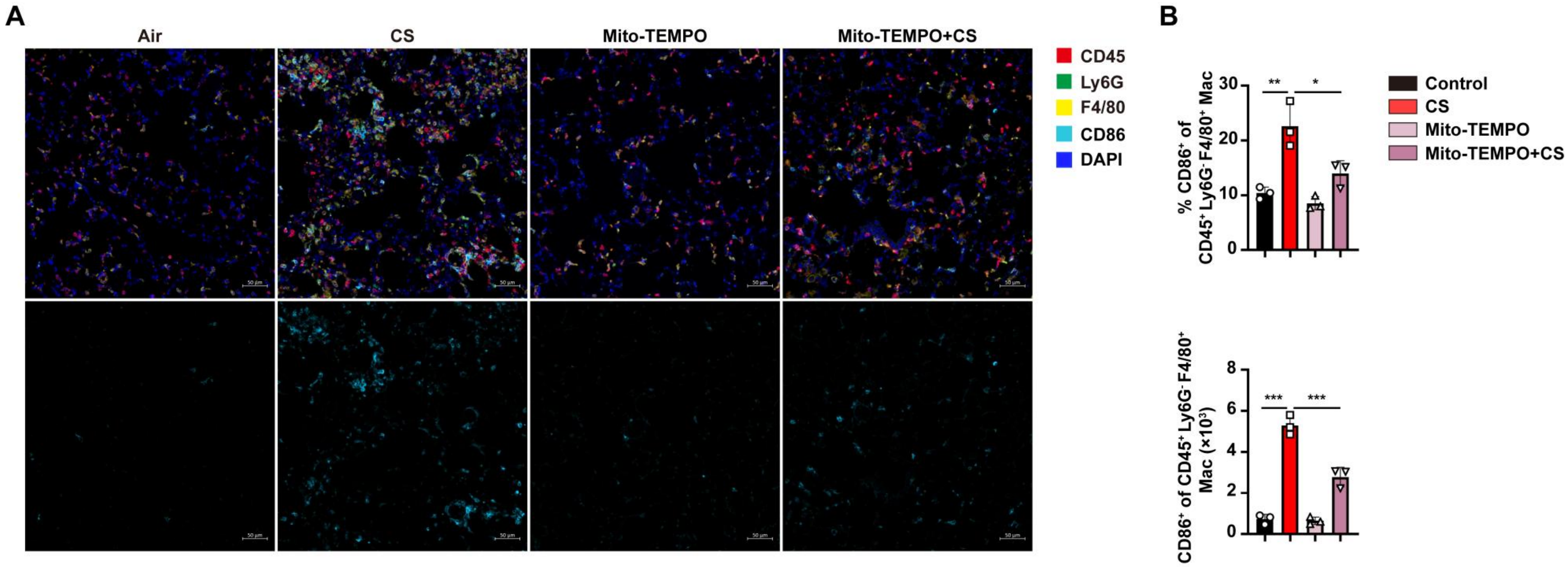

Fig. S11 Mito-TEMPO attenuates CSE-induced inflammatory M1 macrophage
(**A-B**): Multiplexed immunohistochemistry showing the (**A**) percentage and (**B**) absolute counts of CD86^{+} macrophage in mice, in the presence or absence of Mito-TEMPO (n = 3 per group). Data are presented as mean ± SD; * indicates $p < 0.05$, *** indicates $p < 0.001$.

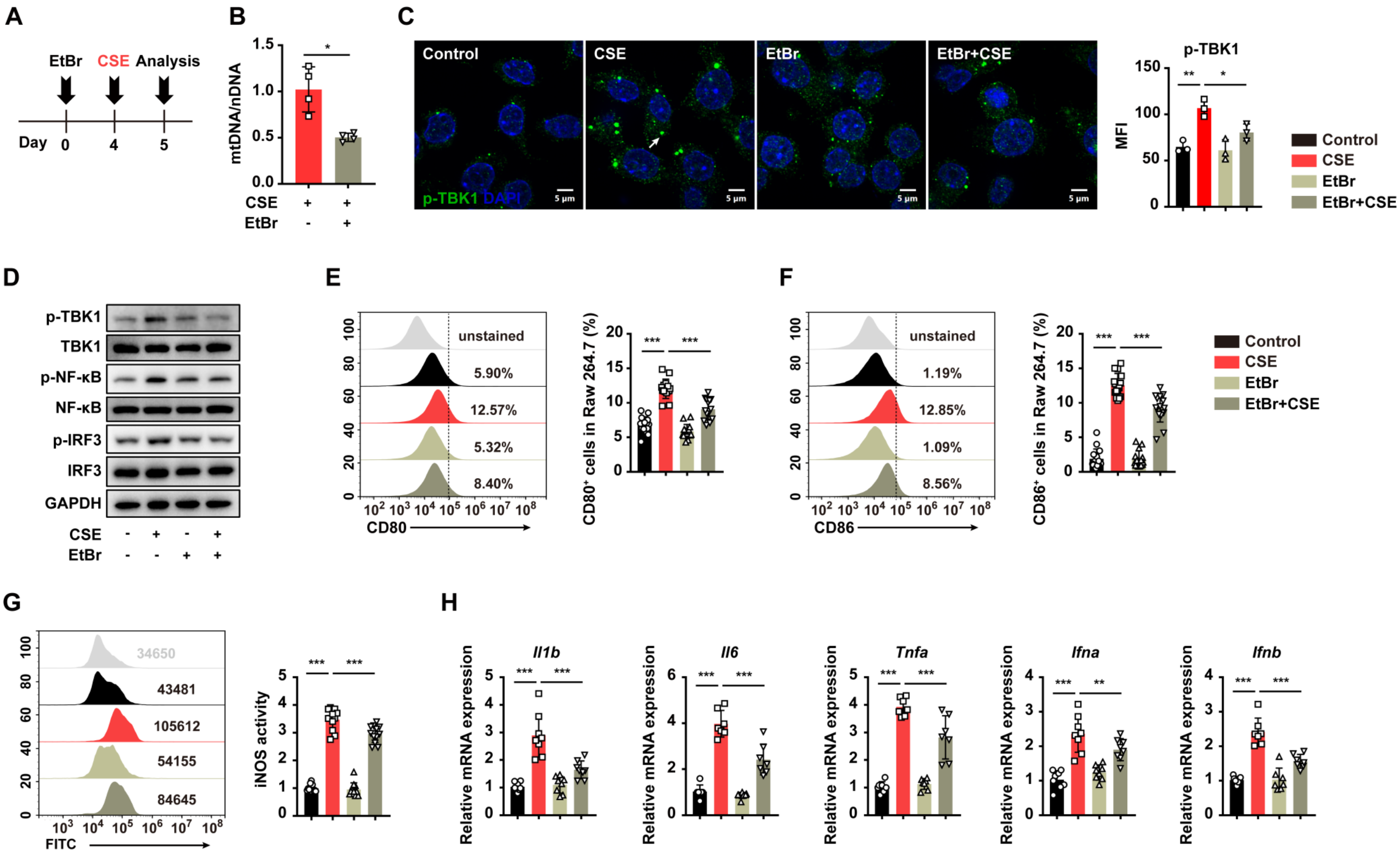

Fig. S12 EtBr-mediated mtDNA depletion drives M1 polarization in CSE-exposed RAW 264.7 cells

(**A**): Schematic of EtBr and CSE treatment strategies in RAW 264.7 cells. (**B**): Quantification of mtDNA copy number (n = 4 per group). (**C**): Immunofluorescence analysis of phosphorylated TBK1 (p-TBK1) (n = 3 per group). (**D**): Western blot analysis of signaling proteins downstream of p-TBK1, p-NF-κB and p-IRF3 (n = 3 per group). (**E-G**): Flow cytometry analysis of the percentage of (**E**) CD80^{+} and (**F**) CD86^{+} cells, alongside (**G**) iNOS activity assays (n = 12-15 per group). (**H**): qPCR analysis of inflammatory cytokines (*Il1b*, *Il6*, *Tnfa*, *Ifna*, and *Ifnb*) mRNA expression levels in RAW 264.7 cells following CSE and EtBr treatment (n = 7-8 per group). Data are presented as mean ± SD; * indicates $p < 0.05$, ** indicates $p < 0.01$, *** indicates $p < 0.001$.

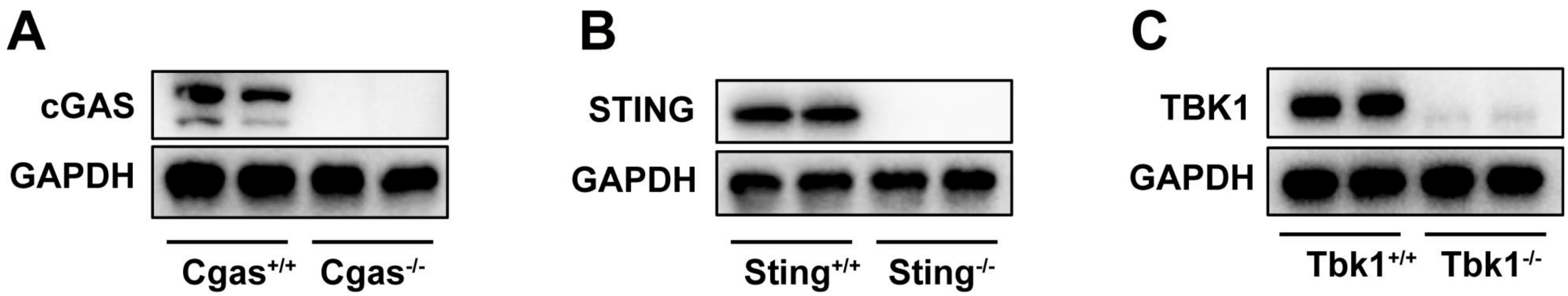

Fig. S13 Western blot validation of cGAS, STING and TBK1 knockout models
(**A-C**): Western blot analysis confirming the absence of cGAS (**A**), STING (**B**) and TBK1 (**C**) protein expression in the respective *Cgas*$^{-/-}$, *Sting*$^{-/-}$, and *Tbk1*$^{-/-}$ in RAW264.7 cell.

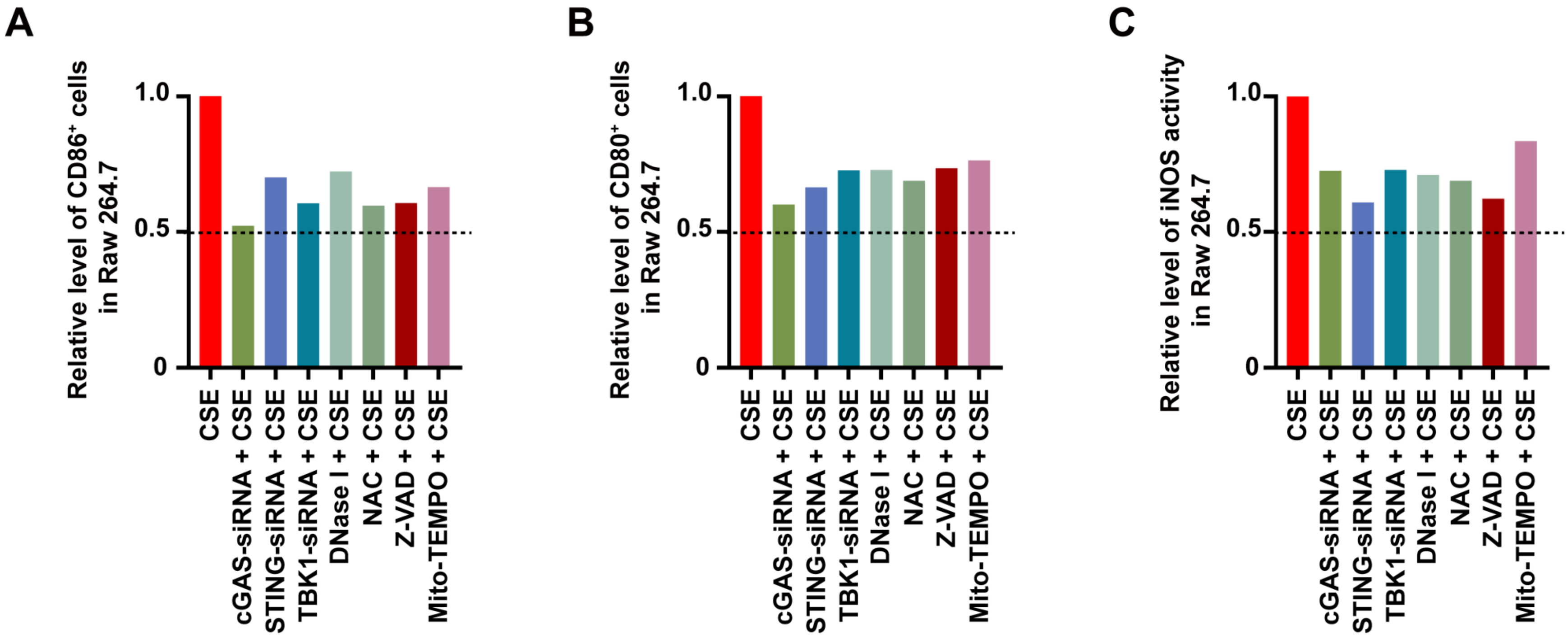

Fig. S14 Comparative efficiency of different treatment strategies on $CD86^+$/$CD80^+$ macrophages and iNOS activity

(**A-C**): Relative levels of $CD86^+$ (**A**) and $CD80^+$ (**B**) macrophages, alongside iNOS (**C**) activity, in RAW 264.7 cell under the indicated treatment conditions.
