# Supplementary material for: Self-DNA mediates tobacco-induced lung inflammation via macrophage-intrinsic cGAS/STING/TBK1 signaling
Source: Cell Commun Signal. 2026 Aug 10;24:607. doi: 10.1186/s12964-026-03124-3 (PMC13761130; doi:10.1186/s12964-026-03124-3)
Supplement: Supplementary file 2 [file 12964_2026_3124_MOESM2_ESM.pdf]

Figure 2C

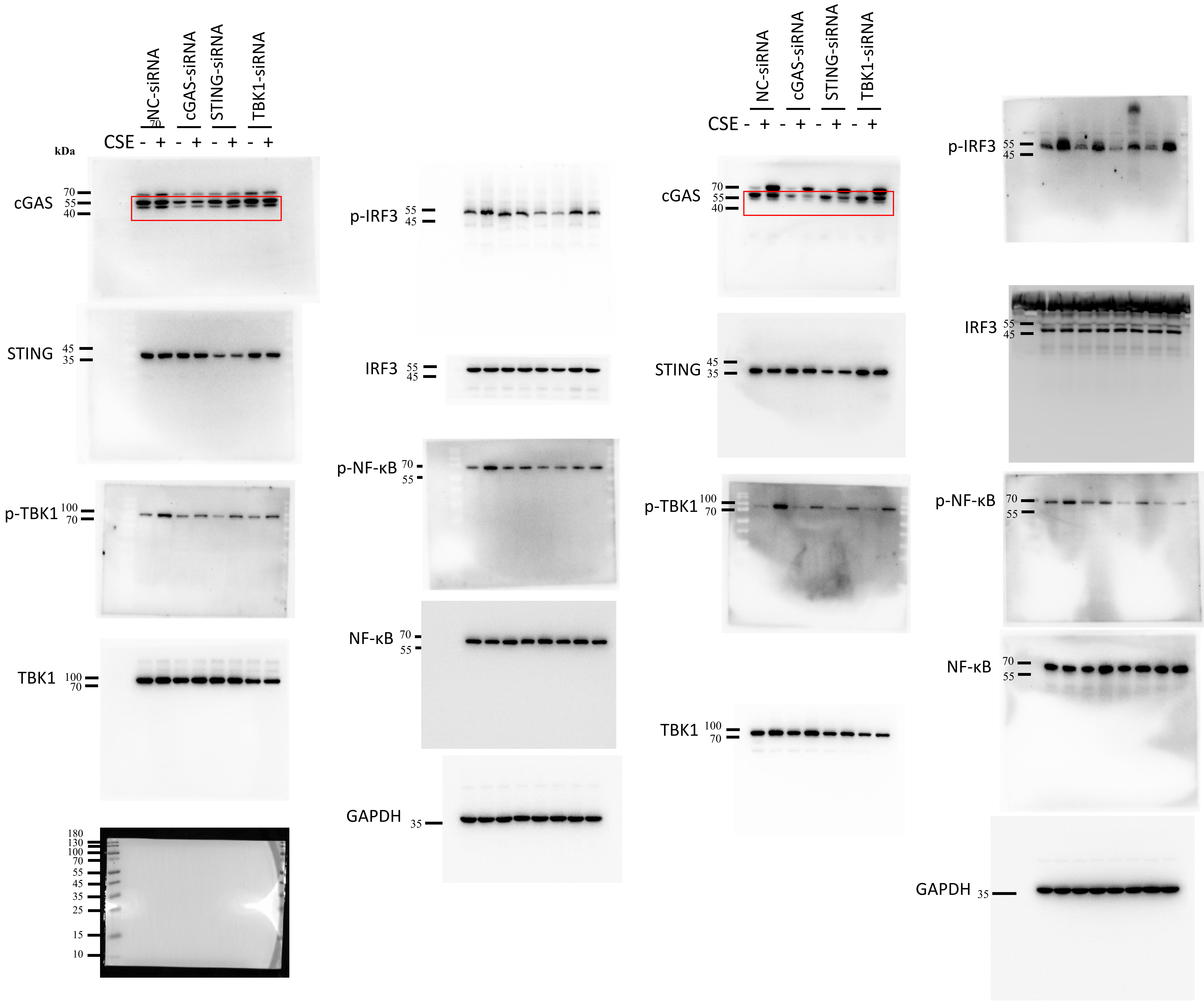

NC-siRNA
cGAS-siRNA
STING-siRNA
TBK1-siRNA
CSE - + - + - + - +
kDa
cGAS
70
55
40
STING
45
35
p-TBK1
100
70
TBK1
100
70
180
130
100
70
55
45
35
25
15
10
p-IRF3
55
45
IRF3
55
45
p-NF-κB
70
55
NF-κB
70
55
GAPDH
35
NC-siRNA
cGAS-siRNA
STING-siRNA
TBK1-siRNA
CSE - + - + - + - +
cGAS
70
55
40
STING
45
35
p-TBK1
100
70
TBK1
100
70
p-IRF3
55
45
IRF3
55
45
p-NF-κB
70
55
NF-κB
70
55
GAPDH
35

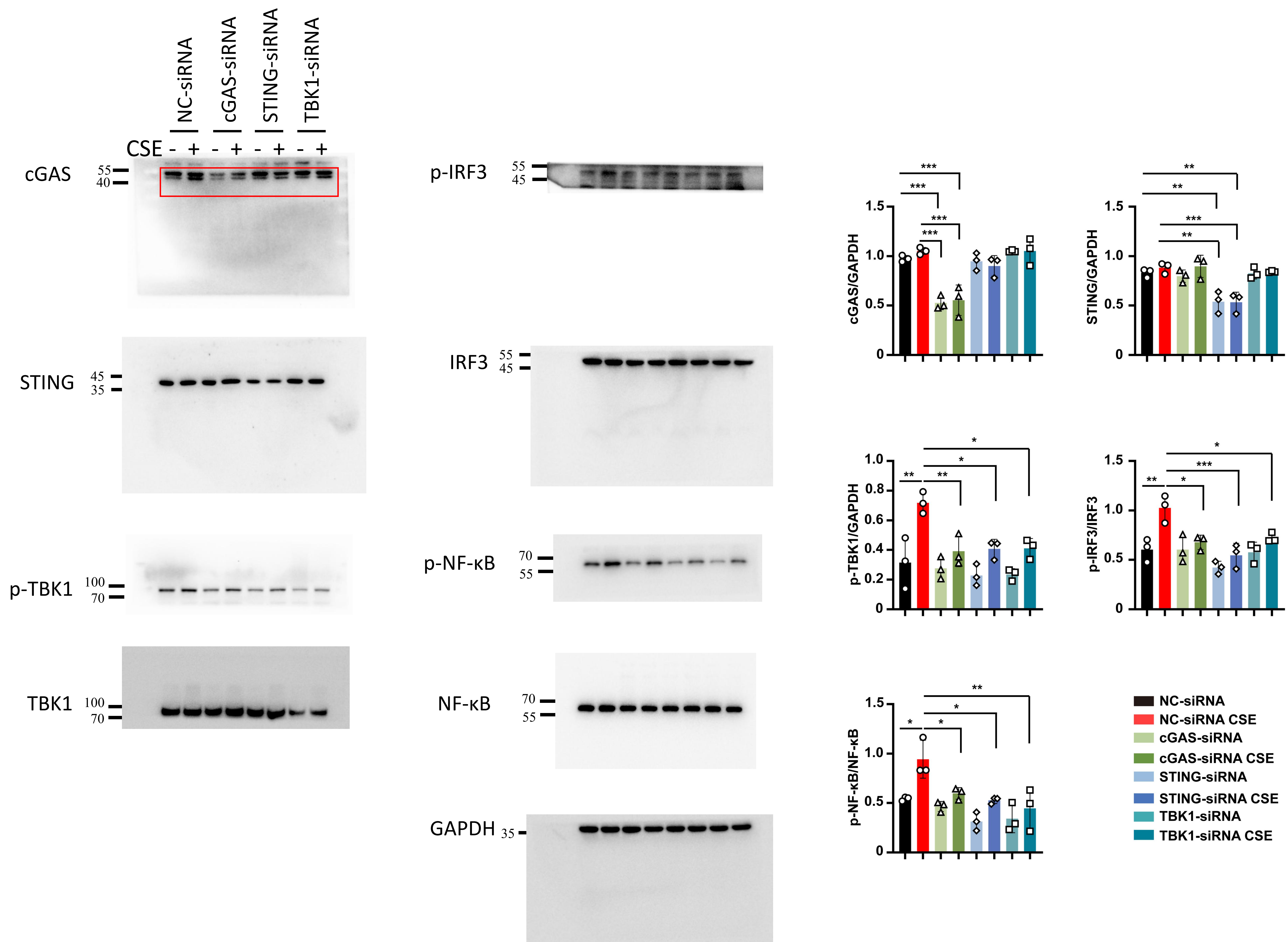

NC-siRNA
cGAS-siRNA
STING-siRNA
TBK1-siRNA
CSE - + - + - + - +
cGAS
55
40
STING
45
35
p-TBK1
100
70
TBK1
100
70
p-IRF3
55
45
IRF3
55
45
p-NF-κB
70
55
NF-κB
70
55
GAPDH
35
cGAS/GAPDH
STING/GAPDH
p-TBK1/GAPDH
p-IRF3/IRF3
p-NF-κB/NF-κB
NC-siRNA
NC-siRNA CSE
cGAS-siRNA
cGAS-siRNA CSE
STING-siRNA
STING-siRNA CSE
TBK1-siRNA
TBK1-siRNA CSE

Figure 4J

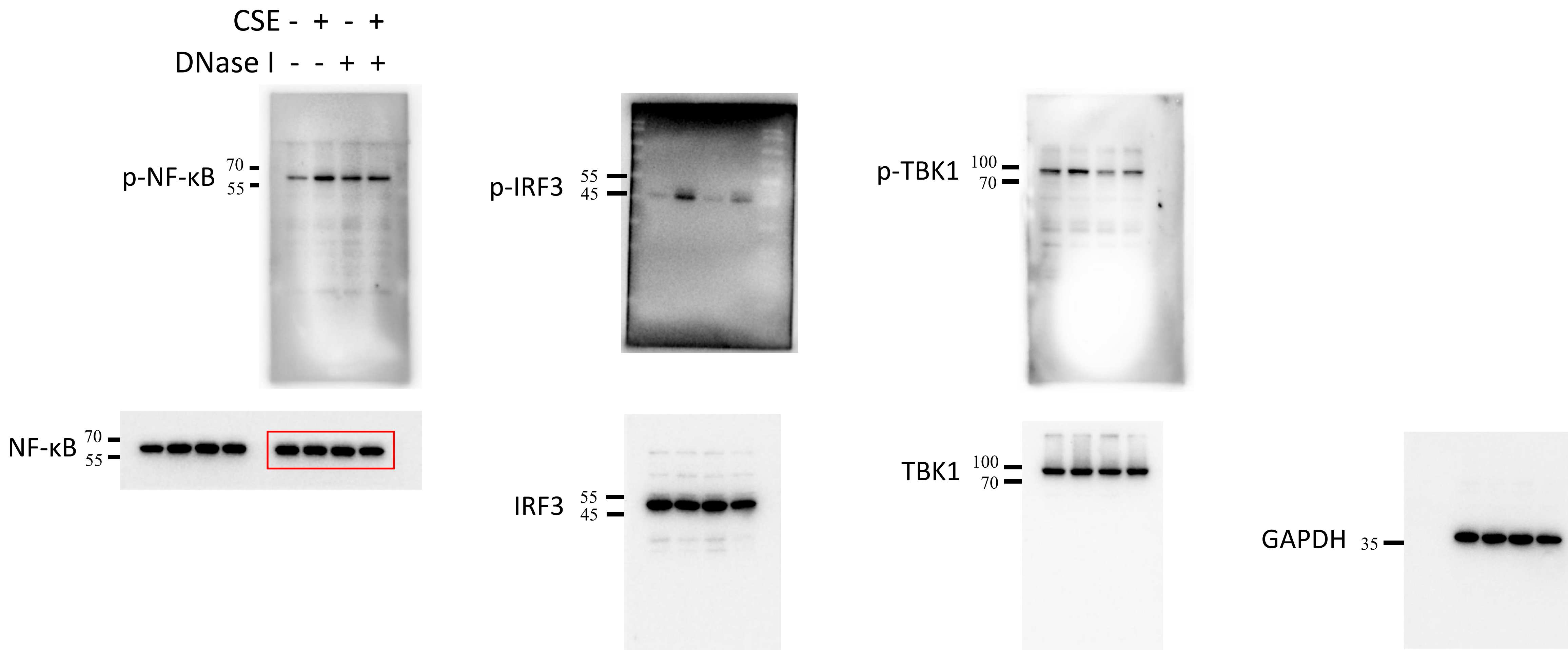
CSE - + - +
DNase I - - + +
p-NF-κB
70
55
p-IRF3
55
45
p-TBK1
100
70
NF-κB
70
55
IRF3
55
45
TBK1
100
70
GAPDH
35

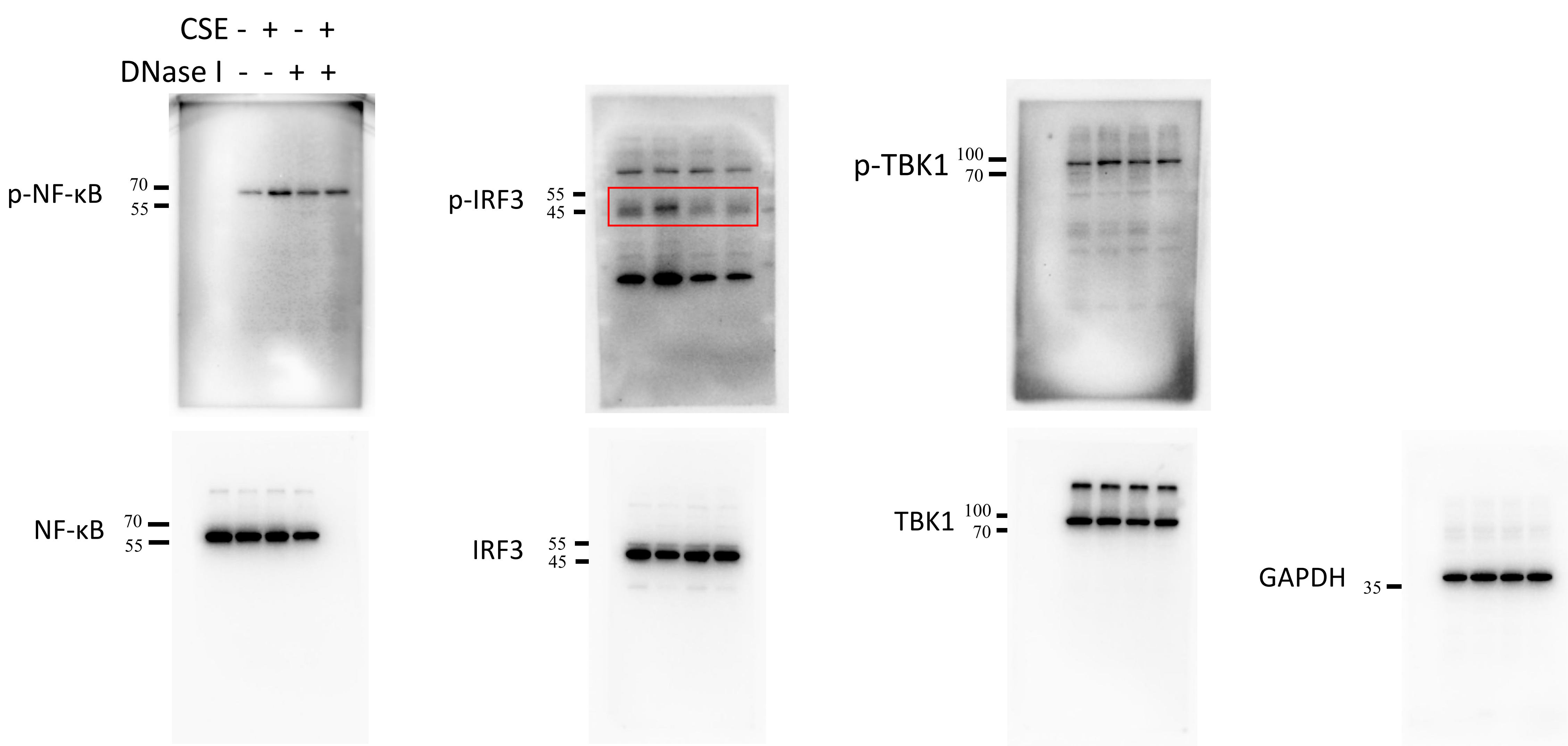
CSE - + - +
DNase I - - + +
p-NF-κB
70
55
p-IRF3
55
45
p-TBK1
100
70
NF-κB
70
55
IRF3
55
45
TBK1
100
70
GAPDH
35

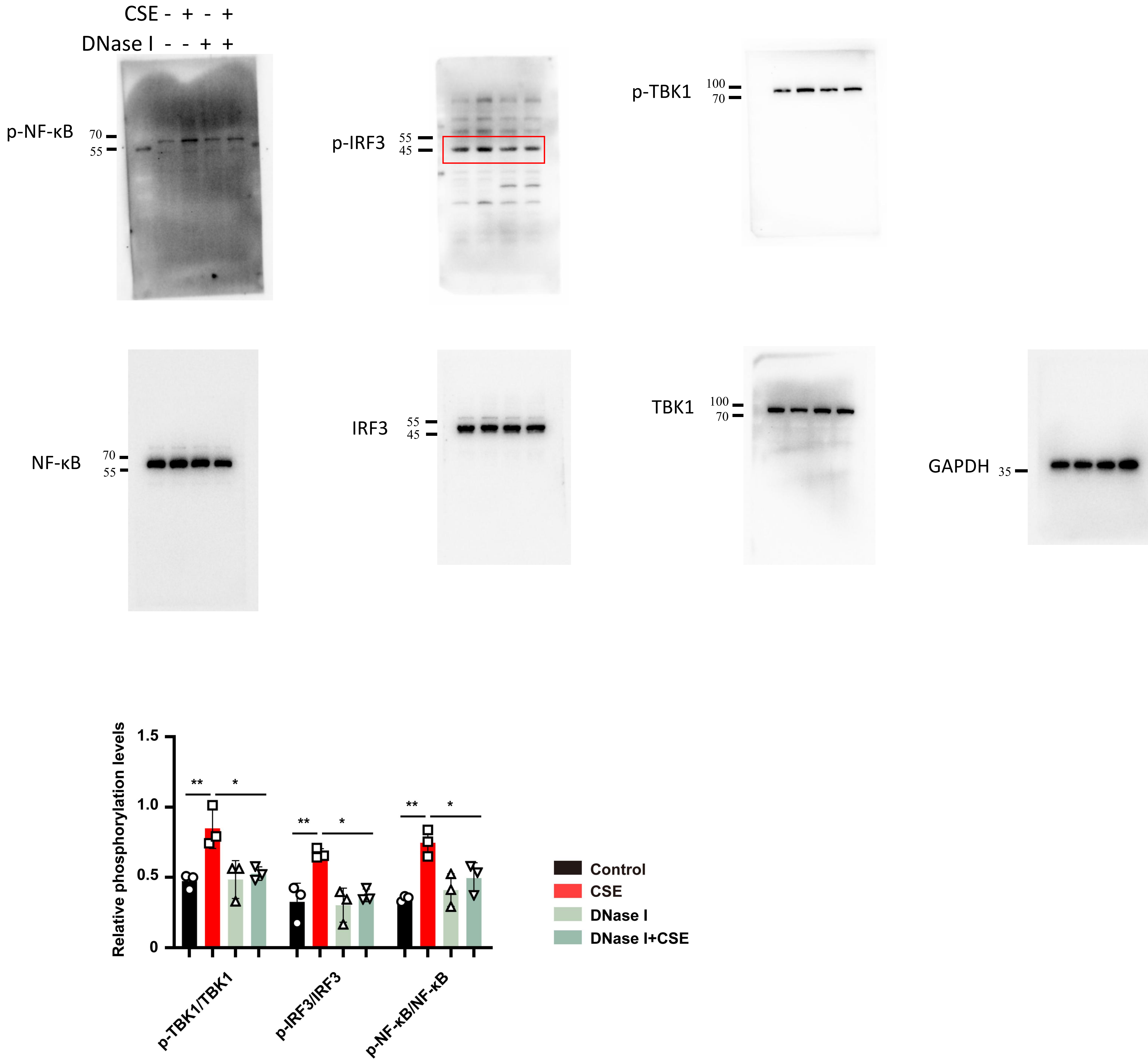

CSE - + - +
DNase I - - + +
p-NF-κB
70
55
p-IRF3
55
45
p-TBK1
100
70
NF-κB
70
55
IRF3
55
45
TBK1
100
70
GAPDH
35
Relative phosphorylation levels
1.5
1.0
0.5
0
**
*
p-TBK1/TBK1
p-IRF3/IRF3
p-NF-κB/NF-κB
Control
CSE
DNase I
DNase I+CSE

Figure 5E

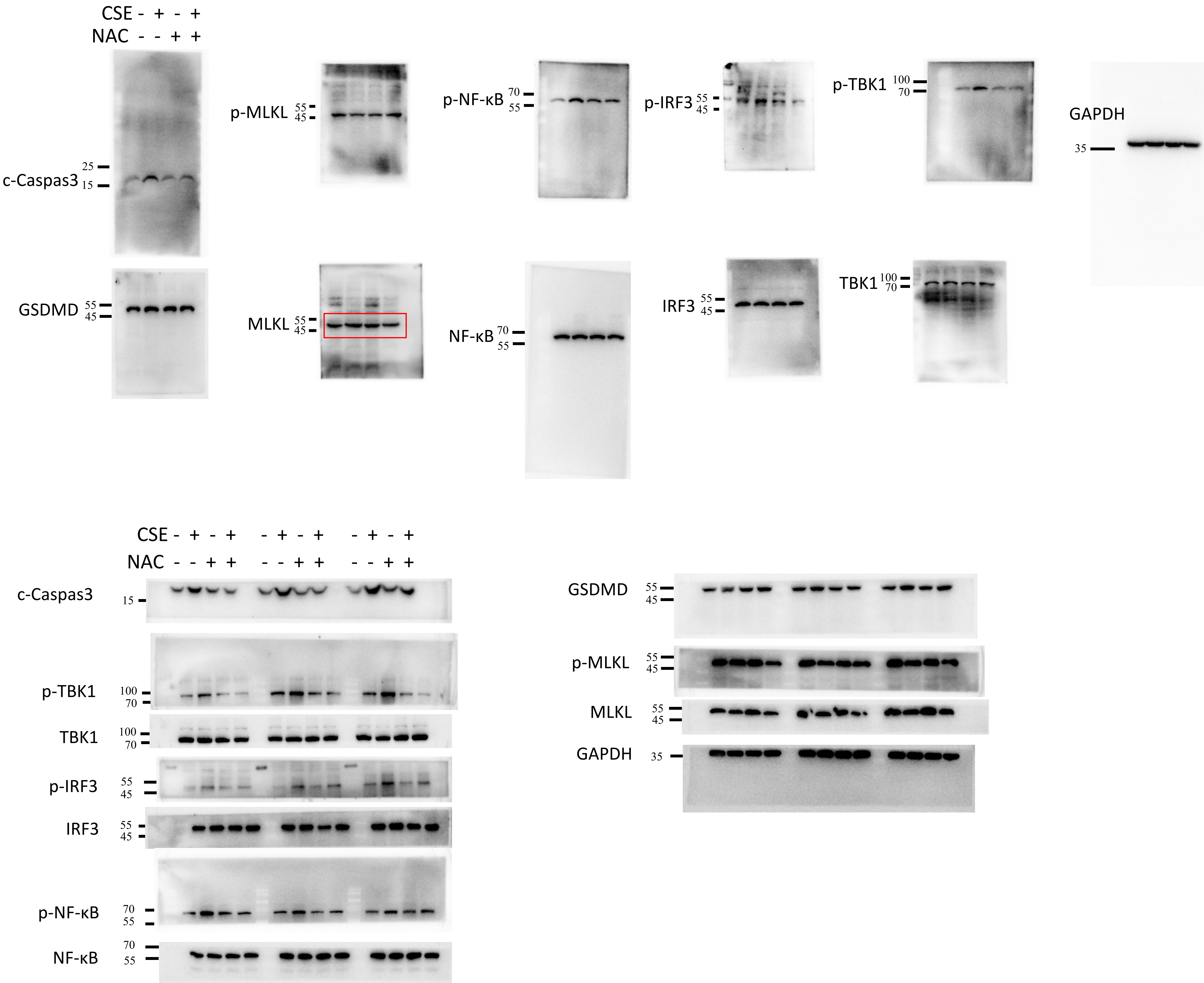
CSE - + - +
NAC - - + +
c-Caspas3
p-MLKL
p-NF-κB
p-IRF3
p-TBK1
GAPDH
GSDMD
MLKL
NF-κB
IRF3
TBK1
CSE - + - + - + - + - + - +
NAC - - + + - - + + - - + +
c-Caspas3
p-TBK1
TBK1
p-IRF3
IRF3
p-NF-κB
NF-κB
GSDMD
p-MLKL
MLKL
GAPDH

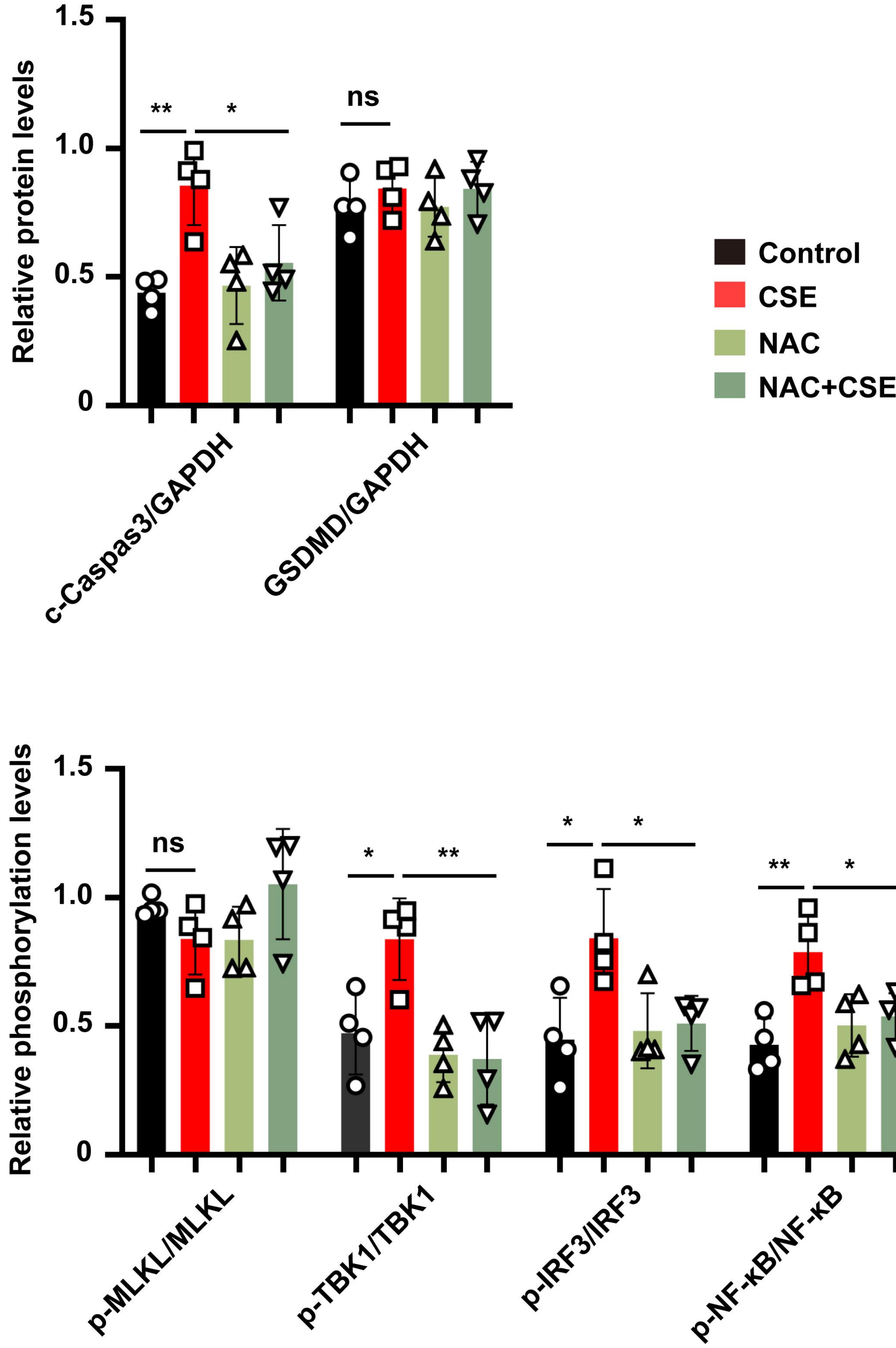
Relative protein levels
1.5
1.0
0.5
0
**
*
ns
c-Caspas3/GAPDH
GSDMD/GAPDH
Control
CSE
NAC
NAC+CSE
Relative phosphorylation levels
1.5
1.0
0.5
0
ns
*
**
*
*
**
*
p-MLKL/MLKL
p-TBK1/TBK1
p-IRF3/IRF3
p-NF-κB/NF-κB

Figure 5L

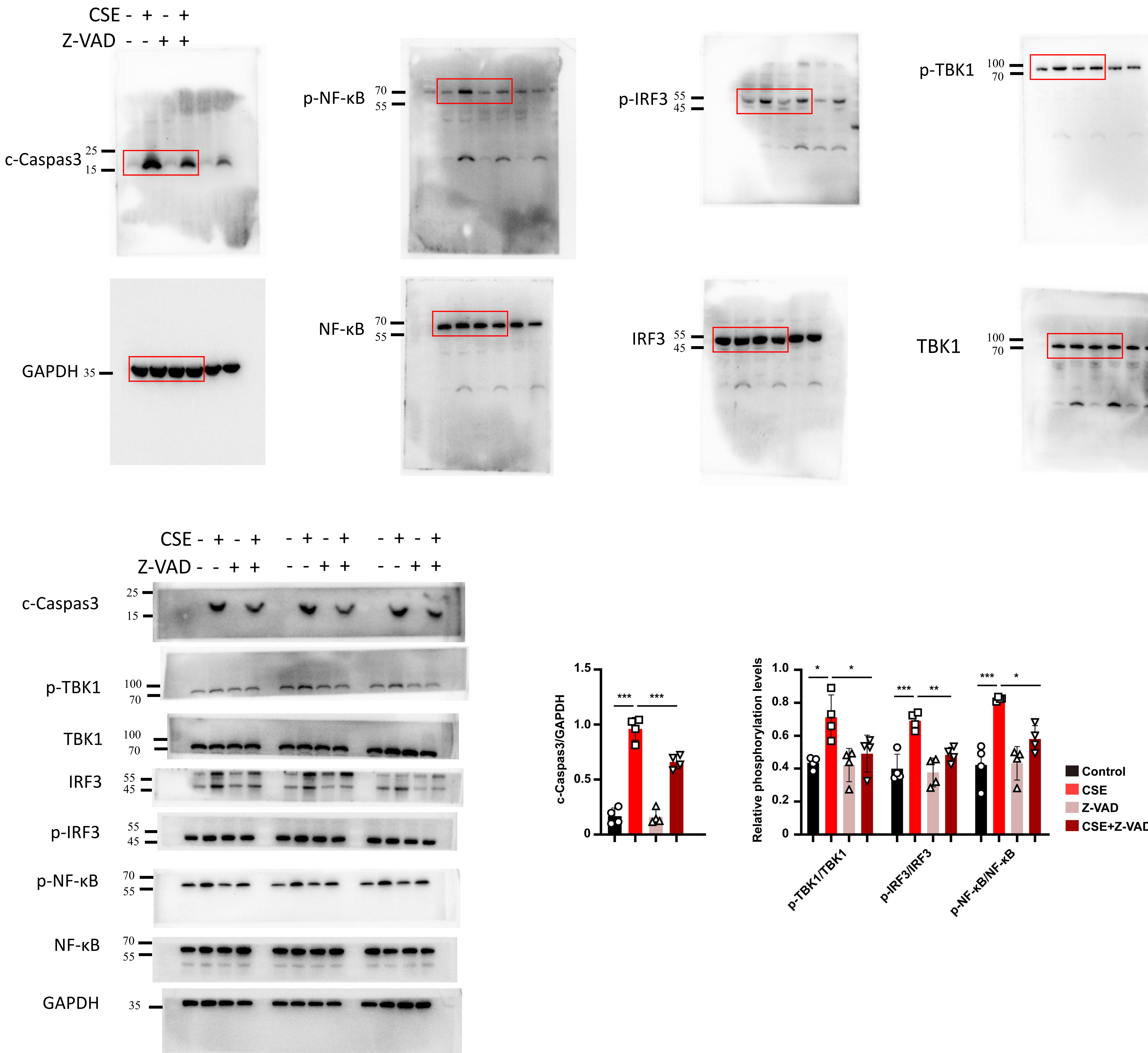

CSE - + - +
Z-VAD - - + +
c-Caspas3
25
15
p-NF-κB
70
55
p-IRF3
55
45
p-TBK1
100
70
GAPDH
35
NF-κB
IRF3
TBK1
p-TBK1/TBK1
p-IRF3/IRF3
p-NF-κB/NF-κB
c-Caspas3/GAPDH
Relative phosphorylation levels
Control
CSE
Z-VAD
CSE+Z-VAD

Figure 6F

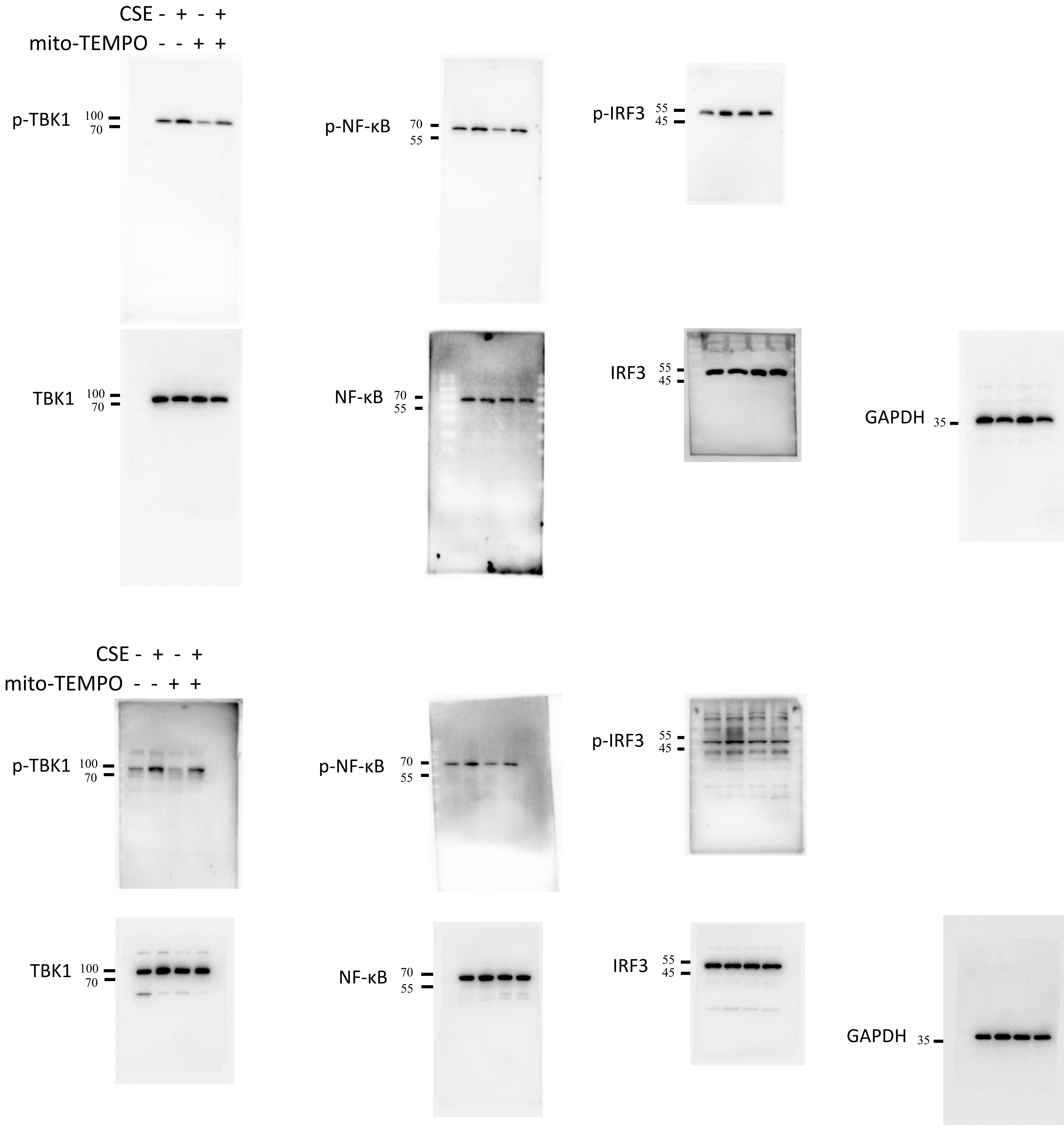
CSE - + - +
mito-TEMPO - - + +
p-TBK1
100
70
p-NF-κB
70
55
p-IRF3
55
45
TBK1
100
70
NF-κB
70
55
IRF3
55
45
GAPDH
35
CSE - + - +
mito-TEMPO - - + +
p-TBK1
100
70
p-NF-κB
70
55
p-IRF3
55
45
TBK1
100
70
NF-κB
70
55
IRF3
55
45
GAPDH
35

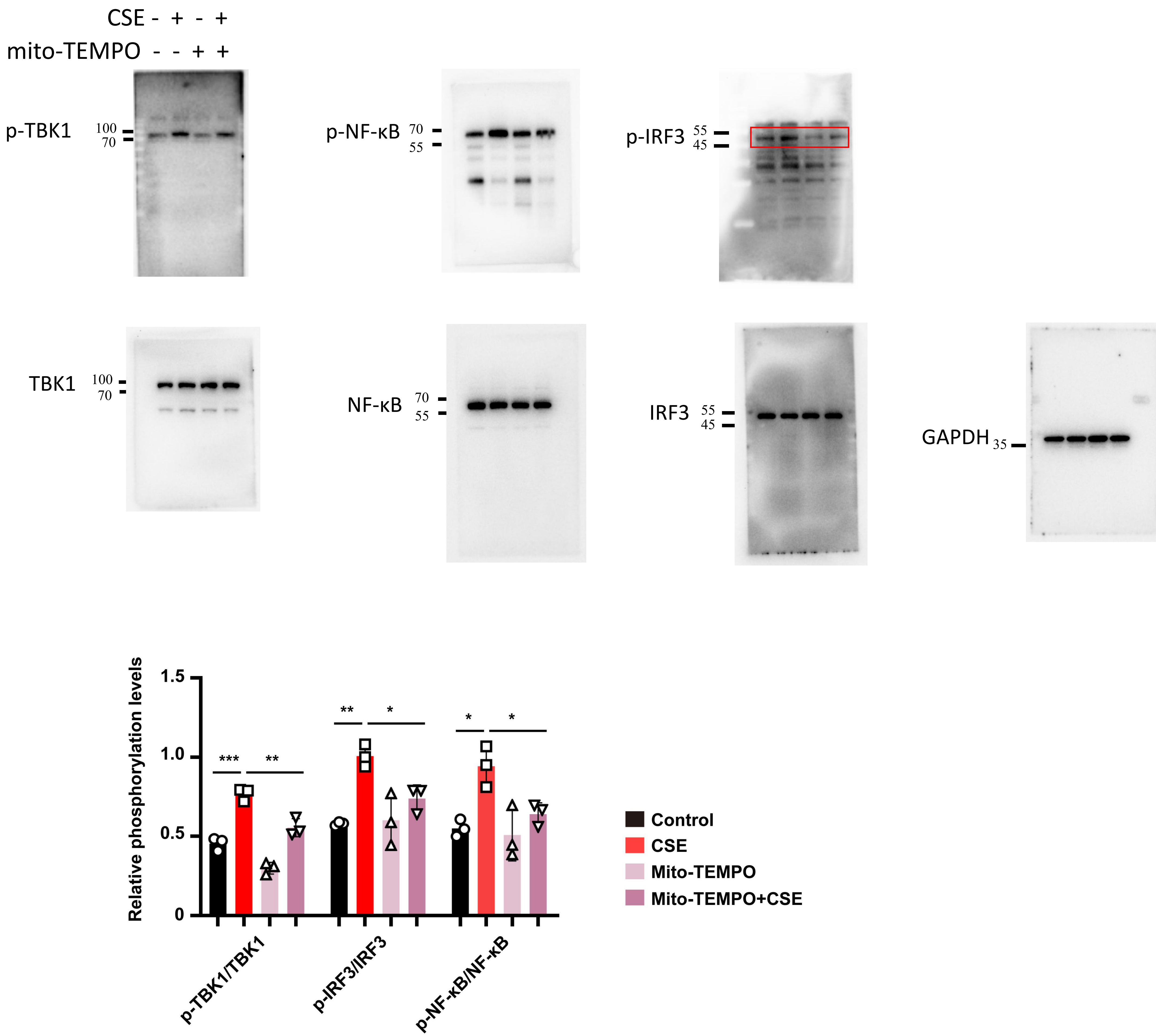
CSE - + - +
mito-TEMPO - - + +
p-TBK1
100
70
p-NF-κB
70
55
p-IRF3
55
45
TBK1
100
70
NF-κB
70
55
IRF3
55
45
GAPDH
35
Relative phosphorylation levels
1.5
1.0
0.5
0
***
**
*
p-TBK1/TBK1
p-IRF3/IRF3
p-NF-κB/NF-κB
Control
CSE
Mito-TEMPO
Mito-TEMPO+CSE

Figure S8B

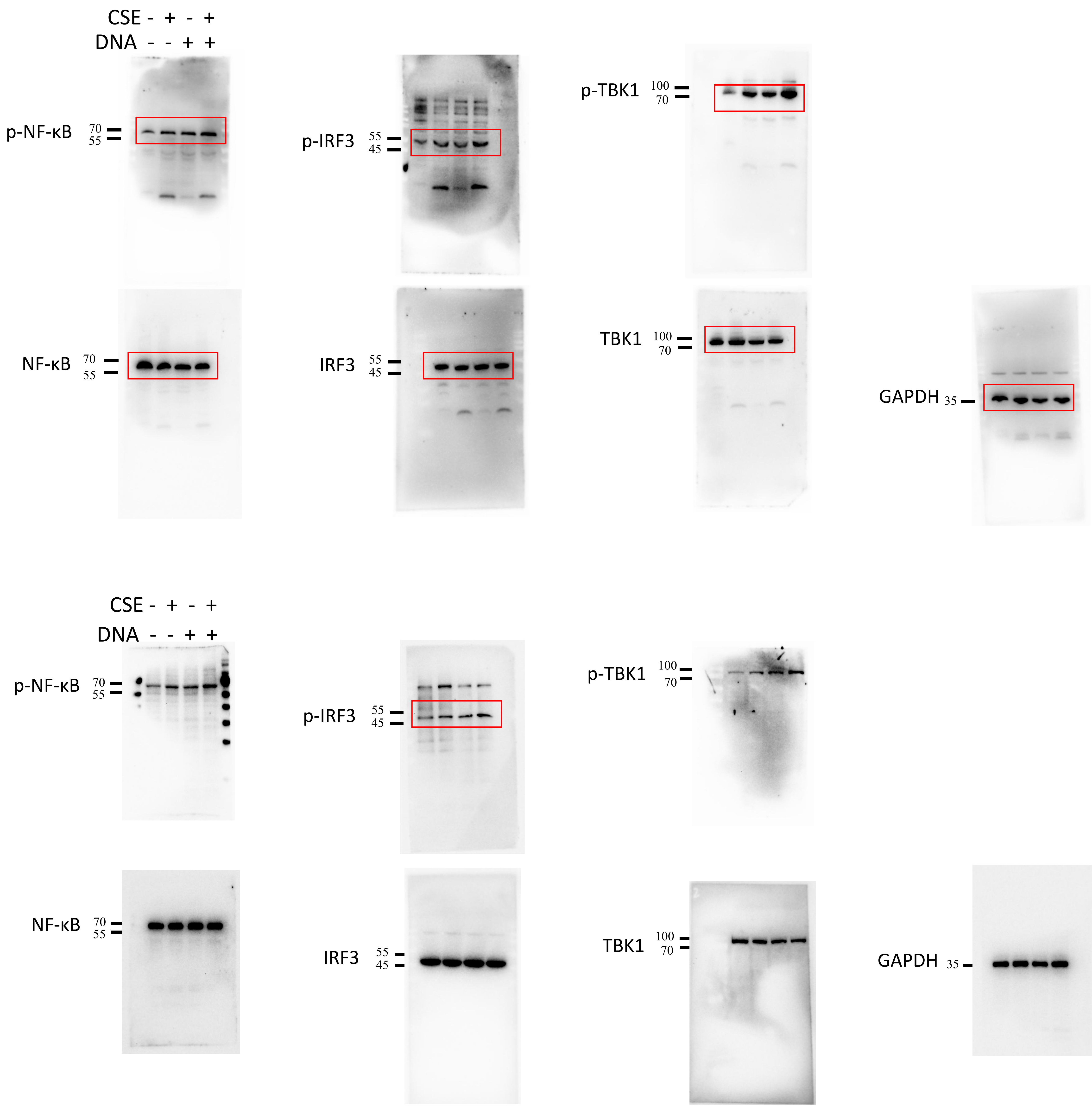
CSE - + - +
DNA - - + +
p-NF-κB
70
55
p-IRF3
55
45
p-TBK1
100
70
NF-κB
70
55
IRF3
55
45
TBK1
100
70
GAPDH
35
CSE - + - +
DNA - - + +
p-NF-κB
70
55
p-IRF3
55
45
p-TBK1
100
70
NF-κB
70
55
IRF3
55
45
TBK1
100
70
GAPDH
35

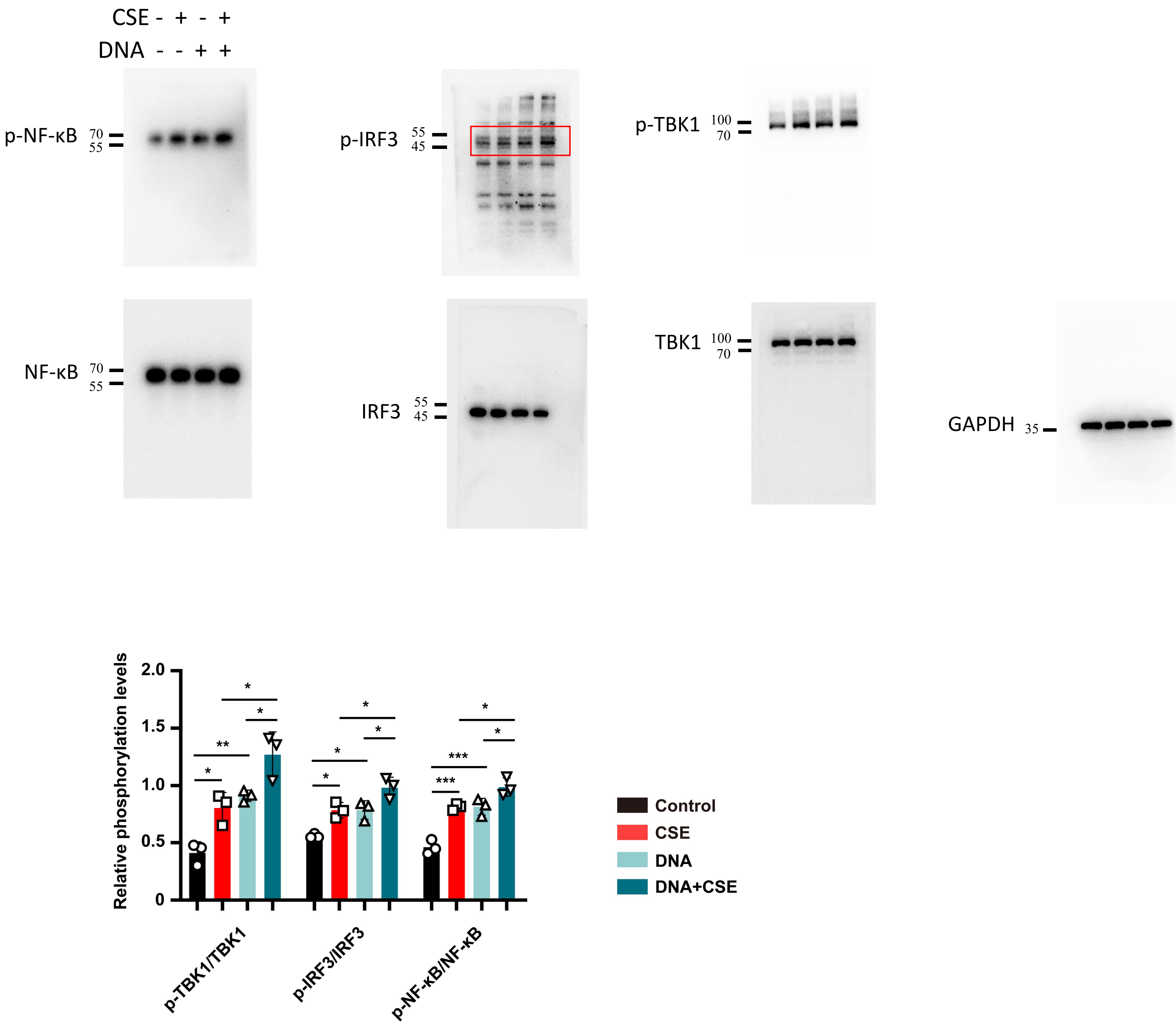
CSE - + - +
DNA - - + +
p-NF-κB 70 55
p-IRF3 55 45
p-TBK1 100 70
NF-κB 70 55
IRF3 55 45
TBK1 100 70
GAPDH 35
Relative phosphorylation levels
2.0
1.5
1.0
0.5
0
p-TBK1/TBK1
p-IRF3/IRF3
p-NF-κB/NF-κB
Control
CSE
DNA
DNA+CSE

Figure S12D

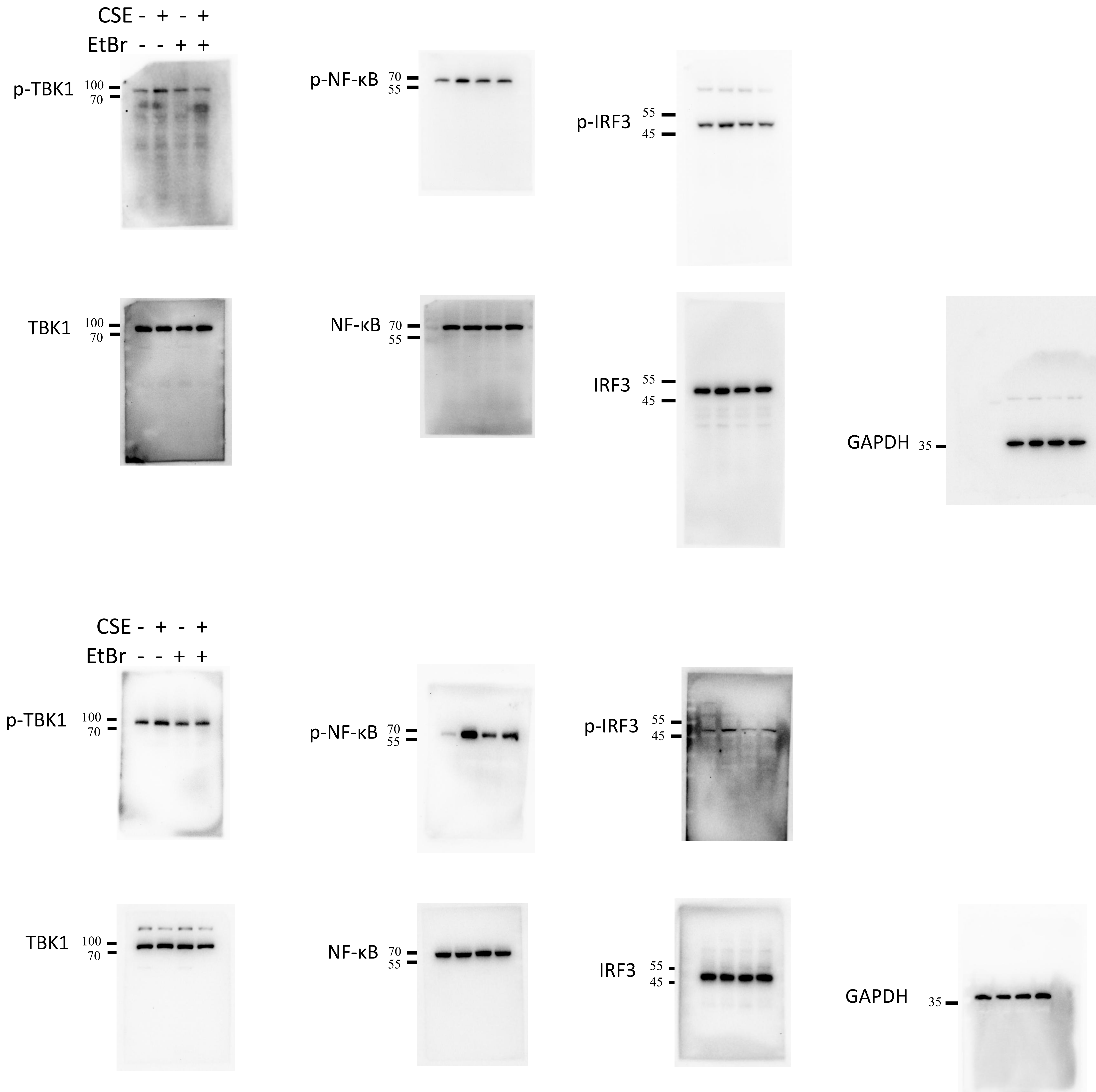

CSE - + - +
EtBr - - + +
p-TBK1
100
70
p-NF-κB
70
55
p-IRF3
55
45
TBK1
100
70
NF-κB
70
55
IRF3
55
45
GAPDH
35
CSE - + - +
EtBr - - + +
p-TBK1
100
70
p-NF-κB
70
55
p-IRF3
55
45
TBK1
100
70
NF-κB
70
55
IRF3
55
45
GAPDH
35

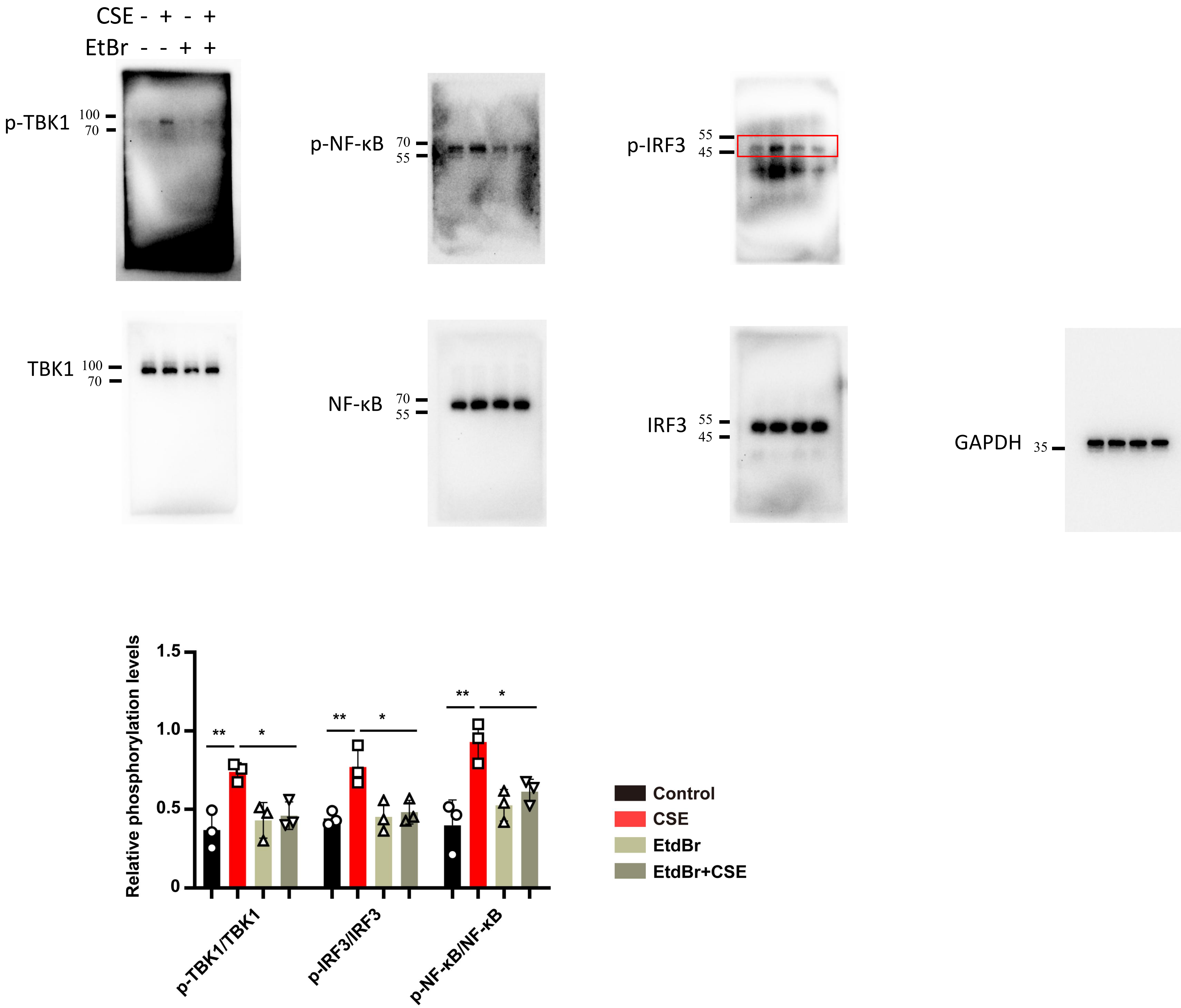

CSE - + - +
EtBr - - + +
p-TBK1
100
70
p-NF-κB
70
55
p-IRF3
55
45
TBK1
100
70
NF-κB
70
55
IRF3
55
45
GAPDH
35
Relative phosphorylation levels
1.5
1.0
0.5
0
**
*
p-TBK1/TBK1
p-IRF3/IRF3
p-NF-κB/NF-κB
Control
CSE
EtdBr
EtdBr+CSE

Figure S13

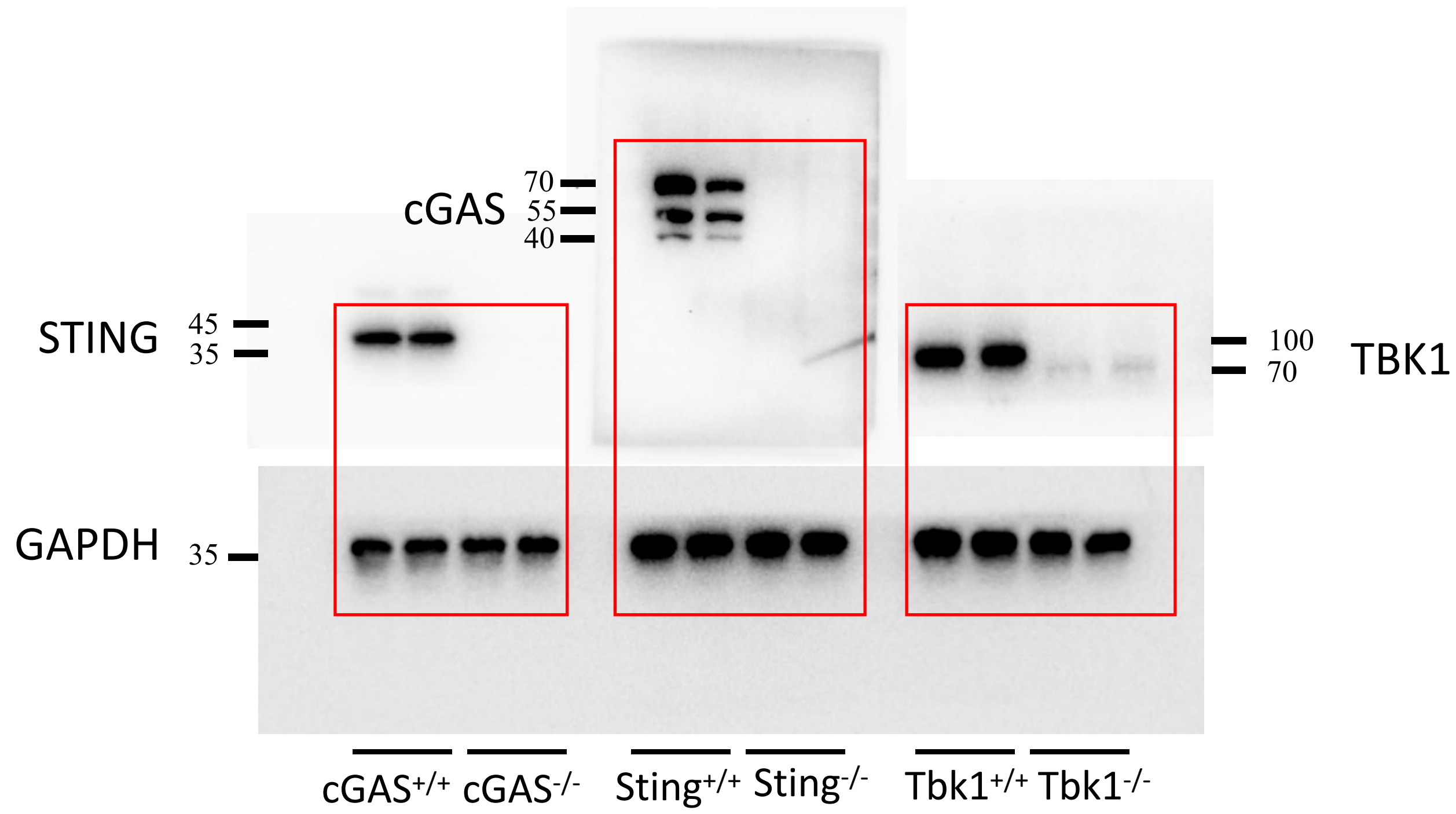

cGAS
70
55
40
STING
45
35
100
70
TBK1
GAPDH
35
cGAS+/+
cGAS-/-
Sting+/+
Sting-/-
Tbk1+/+
Tbk1-/-
